# Supplementary material for: Higher productivity in forests with mixed mycorrhizal strategies
Source: Nat Commun. 2023 Mar 13;14:1377. doi: 10.1038/s41467-023-36888-0 (PMC10011551; doi:10.1038/s41467-023-36888-0)
Supplement: Supplementary file 1 — Supplmentary Information [file 41467_2023_36888_MOESM1_ESM.pdf]

Supplementary Information

Higher productivity in forests with mixed mycorrhizal strategies

Shan Luo[1,2†*], Richard P. Phillips[3†], Insu Jo[4], Songlin Fei[5], Jingjing Liang[5], Bernhard Schmid[6], Nico Eisenhauer[1,2]

[1]German Centre for Integrative Biodiversity Research (iDiv) Halle-Jena-Leipzig, Puschstrasse 4, 04103 Leipzig, Germany

[2]Institute of Biology, Leipzig University, Puschstrasse 4, 04103 Leipzig, Germany

[3]Department of Biology, Indiana University, Bloomington, IN 47405, USA

[4]Manaaki Whenua – Landcare Research, Lincoln 7608, New Zealand

[5]Department of Forestry and Natural Resources, Purdue University, West Lafayette, IN 47907, USA

[6]Department of Geography, Remote Sensing Laboratories, University of Zürich, Winterthurerstrasse 190, CH-8057 Zürich, Switzerland

[†]These authors contributed equally

[*]Correspondence: Shan Luo (luoshan.hi@gmail.com)

Supplementary Table S1 | Summary of ANOVA results for forest productivity (log-transformed). The explanatory variables include ecoregions, AM proportion (linear and quadratic terms), tree species richness (log-transformed), interactions between AM proportion and richness, interactions between AM proportion and ecoregions, stand age, elevation, slope, climatic variables (mean annual temperature, mean annual precipitation and temperature seasonality), and soil pH. Overall analysis (Model A) followed by separate analyses for low-richness (five or less tree species; Model B) and high-richness (more than five species; Model C) plots. Key to abbreviations: df, degree of freedom; %SS, percentage of the total sum of squares explained by each term; *F*, variance ration; *P*, probability of Type I error.

	Forest productivity				
Source of variation	df	Mean square	%SS	*F*	*P*
Model A: overall model					
Ecoregion	34	1940	46.19	2767.08	<0.001
Linear AM proportion (AM)	1	2231	1.56	3182.21	<0.001
Quadratic AM proportion (AM2)	1	2283	1.60	3255.84	<0.001
Log richness (SR)	1	13762	9.64	19628.47	<0.001
AM × SR	1	653	0.46	931.64	<0.001
AM2 × SR	1	6	0.01	8.79	0.003
AM × ecoregion	34	40	0.96	57.22	<0.001
AM2 × ecoregion	34	17	0.40	24.25	<0.001
Stand age	1	2708	1.90	3862.92	<0.001
Elevation	1	142	0.10	202.98	<0.001
Slope	1	15	0.01	21.30	<0.001
Mean annual temperature	1	2	0.00	3.53	0.060
Mean annual precipitation	1	749	0.07	1067.92	<0.001
Temperature seasonality	1	101	0.03	143.73	<0.001
Soil pH	1	40	0.04	57.24	<0.001
Residuals	74448	1	36.55		
Model B: low richness					
Ecoregion	34	1150.2	37.60	1074.73	<0.001
Linear AM proportion (AM)	1	2578.5	2.48	2409.40	<0.001
Quadratic AM proportion (AM2)	1	780.9	0.75	729.65	<0.001
AM × ecoregion	34	69.7	2.28	65.14	<0.001
AM2 × ecoregion	34	16.6	0.54	15.52	<0.001
Stand age	1	1356.0	1.30	1267.08	<0.001

Elevation	1	241.0	0.23	225.15	<0.001
Slope	1	22.6	0.02	21.11	<0.001
Mean annual temperature	1	47.1	0.05	43.98	<0.001
Mean annual precipitation	1	1382.7	1.33	1292.01	<0.001
Temperature seasonality	1	87.9	0.08	82.15	<0.001
Soil pH	1	157.9	0.15	147.59	<0.001
Residuals	51665	1.1	53.17		
Model C: high richness					
Ecoregion	30	43.7	16.51	172.09	<0.001
Linear AM proportion (AM)	1	1.7	0.03	6.65	0.010
Quadratic AM proportion (AM2)	1	0.1	0.00	0.52	0.471
AM × ecoregion	28	2.1	0.74	8.37	<0.001
AM2 × ecoregion	28	1.1	0.38	4.24	<0.001
Stand age	1	705.2	8.89	2780.03	<0.001
Elevation	1	8.9	0.11	35.10	<0.001
Slope	1	0.0	0.00	0.01	0.492
Mean annual temperature	1	6.3	0.08	24.90	<0.001
Mean annual precipitation	1	11.9	0.15	46.98	<0.001
Temperature seasonality	1	40.7	0.52	160.47	<0.001
Soil pH	1	4.6	0.05	17.97	0.011
Residuals	22690	0.3	72.54		

Supplementary Table S2 | Summary of ANOVA results for forest productivity (log-transformed) when using different thresholds for splitting richness levels. Model A1 & A2, analyses for plots with low (richness ≤ 4) vs. high species richness (richness > 4); Model B1 & B2, analyses for plots with low (richness ≤ 6) vs. high species richness (richness > 6); Model C1-C3, analyses for plots with low (richness ≤ 6) vs. intermediate (6 < richness ≤ 12) vs. high species richness (richness >12). Results of model C1 are the same as that of model B1 and therefore not presented. Key to abbreviations: df, degree of freedom; *F*, variance ration; *P*, probability of Type I error.

	Forest productivity			
Source of variation	df	Mean square	*F*	*P*
Model A1: low (richness ≤ 4)				
Ecoregion	34	910.8	802.13	<0.001
Linear AM proportion (AM)	1	2628.2	2314.68	<0.001
Quadratic AM proportion (AM2)	1	450.5	396.78	<0.001
AM × ecoregion	34	63.0	55.49	<0.001
AM2 × ecoregion	34	14.0	12.34	<0.001
Stand age	1	1218.2	1072.89	<0.001
Elevation	1	232.9	205.10	<0.001
Slope	1	7.6	6.66	0.009
Mean annual temperature	1	73.6	64.81	<0.001
Mean annual precipitation	1	1298.3	1143.43	<0.001
Temperature seasonality	1	74.1	65.25	<0.001
Soil pH	1	146.2	128.78	<0.001
Residuals	44299	1.1		
Model A2: high (richness > 4)				
Ecoregion	32	78.5	262.28	<0.001
Linear AM proportion (AM)	1	10.8	36.22	<0.001
Quadratic AM proportion (AM2)	1	2.0	6.73	0.009
AM × ecoregion	29	3.7	12.38	<0.001
AM2 × ecoregion	29	2.1	6.87	<0.001
Stand age	1	970.8	3243.60	<0.001
Elevation	1	9.7	32.37	<0.001
Slope	1	1.2	3.95	0.047
Mean annual temperature	1	16.9	55.59	<0.001

Mean annual precipitation	1	22.5	75.13	<0.001
Temperature seasonality	1	48.5	162.13	<0.001
Soil pH	1	1.5	4.95	0.026
Residuals	30052	0.3		
Model B1: low (richness ≤ 6)				
Ecoregion	34	1362.6	1334.12	<0.001
Linear AM proportion (AM)	1	2483.9	2446.55	<0.001
Quadratic AM proportion (AM2)	1	1134.1	1117.09	<0.001
AM × ecoregion	34	73.5	72.38	<0.001
AM2 × ecoregion	34	17.9	17.62	<0.001
Stand age	1	1429.6	1408.11	<0.001
Elevation	1	238.9	235.32	<0.001
Slope	1	33.7	33.15	<0.001
Mean annual temperature	1	35.6	35.09	<0.001
Mean annual precipitation	1	1415.9	1394.58	<0.001
Temperature seasonality	1	88.7	87.33	<0.001
Soil pH	1	158.1	155.69	<0.001
Residuals	58022	1.0		
Model B2: high (richness > 6)				
Ecoregion	27	26.7	123.00	<0.001
Linear AM proportion (AM)	1	0.1	0.58	0.447
Quadratic AM proportion (AM2)	1	0.1	0.49	0.484
AM × ecoregion	23	1.4	6.60	<0.001
AM2 × ecoregion	19	0.6	2.73	<0.001
Stand age	1	525.8	244.83	<0.001
Elevation	1	6.7	30.66	<0.001
Slope	1	0.6	2.90	0.089
Mean annual temperature	1	2.6	12.06	<0.001
Mean annual precipitation	1	6.0	27.70	<0.001
Temperature seasonality	1	31.6	145.48	<0.001
Soil pH	1	3.9	17.94	<0.001
Residuals	16350	0.2		
Model C1: low (richness ≤ 6)				
Model C2: intermediate (6 < richness ≤ 12)				
Ecoregion	27	26.2	119.79	<0.001
Linear AM proportion (AM)	1	0.4	1.65	0.198
Quadratic AM proportion(AM2)	1	0.2	1.11	0.293
AM × ecoregion	23	1.4	6.52	<0.001
AM2 × ecoregion	19	0.6	2.77	<0.001
Stand age	1	509.7	2331.34	<0.001
Elevation	1	6.8	31.11	<0.001
Slope	1	0.7	3.34	0.068

Mean annual temperature	1	2.7	12.30	<0.001
Mean annual precipitation	1	5.1	23.50	<0.001
Temperature seasonality	1	30.4	139.20	<0.001
Soil pH	1	4.2	19.07	<0.001
Residuals	15897	0.2		
Model C3: high (richness > 12)				
Ecoregion	10	0.9	7.21	<0.001
Linear AM proportion (AM)	1	0.4	3.21	0.074
Quadratic AM proportion(AM2)	1	0.1	1.14	0.286
AM × ecoregion	9	0.3	2.33	0.014
AM2 × ecoregion	8	0.1	1.18	0.311
Stand age	1	17.2	137.56	<0.001
Elevation	1	0.0	0.38	0.539
Slope	1	0.4	2.94	0.087
Mean annual temperature	1	0.1	1.14	0.287
Mean annual precipitation	1	0.5	3.90	0.049
Temperature seasonality	1	0.8	6.11	0.014
Soil pH	1	0.0	0.18	0.674
Residuals	416	0.1		

Supplementary Table S3 | Mean tree species richness (± SE) at each ecoregion. 'N', number of plots.

Ecoregion	N	Richness
American Semi-Desert and Desert	122	1.74 ± 0.07
Arizona - New Mexico Mountains Semi-Desert - Open Woodland[*]	1544	2.61 ± 0.03
Black Hills Coniferous Forest[*]	140	1.69 ± 0.06
California Coastal Chaparral Forest and Shrub	56	2.95 ± 0.21
California Coastal Range Open Range Open Woodland - Shrub	42	1.90 ± 0.14
California Coastal Steppe - Mixed Forest - Redwood Forest	257	3.33 ± 0.08
Cascade Mixed Forest - Coniferous Forest - Alpine Meadow	2390	2.90 ± 0.03
Central Appalachian Broadleaf Forest-Coniferous Forest-Meadow	3613	6.96 ± 0.04
Central Interior Broadleaf Forest	4702	6.55 ± 0.04
Chihuahuan Semi-Desert[*]	1150	1.50 ± 0.03
Colorado Plateau Semi-Desert	2936	2.08 ± 0.02
East Laurentian Mixed Forest	2947	5.44 ± 0.04
Eastern Broadleaf Forest (Oceanic)	4146	6.25 ± 0.04
Everglades	61	2.98 ± 0.24
Great Plains - Palouse Dry Steppe	961	1.83 ± 0.03
Great Plains Steppe	261	2.68 ± 0.09
Intermountain Semi-Desert[*]	581	1.33 ± 0.03
Intermountain Semi-Desert and Desert	1653	1.77 ± 0.02
Lower Mississippi Riverine Forest	915	5.28 ± 0.08
Middle Rocky Mountains Steppe - Coniferous Forest - Alpine	2678	2.26 ± 0.02
Midwest Broadleaf Forest	1891	4.44 ± 0.05
Nevada-Utah Mountains Semi-Desert - Coniferous Forest - Alpine Meadow	1935	2.13 ± 0.02
New England-Adirondack	2424	5.44 ± 0.04
Northern Rocky Mountains Steppe - Coniferous Forest - Alpine Meadow	2121	3.25 ± 0.03
Ouachita Mixed Forest-Meadow	534	5.61 ± 0.10
Outer Coastal Plain Mixed Forest	7399	5.11 ± 0.03
Pacific Lowland Mixed Forest	226	3.19 ± 0.10
Prairie Parkland (Subtropical)	2166	3.25 ± 0.04
Prairie Parkland (Temperate)	1454	4.92 ± 0.07
Sierren Steppe - Mixed Forest - Coniferous Alpine Meadow	2433	2.93 ± 0.03
Southeastern Mixed Forest	8096	6.46 ± 0.03
Southern Rocky Mountain Steppe - Open Woodland - Coniferou	3520	2.37 ± 0.02
Southern Rocky Mountain Steppe - Open Woodland - Coniferous Forest - Alpine Meadow	428	1.93 ± 0.04
Southwest Plateau and Plains Dry Steppe and Shrub	3629	1.63 ± 0.01
West Laurentian Mixed Forest	5152	4.34 ± 0.03

[*] Ecoregions with significant linear-negative relationships between AM proportion and productivity.

Supplementary Table S4 | Ten of the most abundant species in ecoregions with significant linear-negative relationships between AM proportion and productivity. 'Basal area' is summed basal area per species per ecoregion.

Ecoregion	Species	Mycorrhiza	Basal area
Arizona - New Mexico Mountains Semi-Desert - Open Woodland	*Pinus ponderosa*	ECM	7398.14
	Juniperus monosperma	AM	5436.42
	Juniperus deppeana	AM	3580.39
	Pinus edulis	ECM	2887.15
	Pseudotsuga menziesii	ECM	1338.55
	Juniperus osteosperma	AM	1202.33
	Quercus gambelii	ECM	1010.32
	Quercus arizonica	ECM	705.71
	Quercus grisea	ECM	660.43
	Abies concolor	ECM	558.94
Black Hills Coniferous Forest	*Pinus ponderosa*	ECM	1928.89
	Quercus macrocarpa	ECM	105.89
	Picea glauca	ECM	89.67
	Juniperus scopulorum	AM	38.02
	Populus tremuloides	ECM	30.42
	Populus deltoides ssp. monilifera	AM+ECM	9.53
	Betula papyrifera	ECM	8.39
	Fraxinus pennsylvanica	AM	5.88
	Acer negundo	AM	3.83
	Ulmus pumila	AM	1.73
Chihuahuan Semi-Desert	*Prosopis glandulosa*	AM	944.52
	Prosopis velutina	AM	935.44
	Juniperus pinchotii	AM	749.31
	Juniperus ashei	AM	676.78
	Quercus arizonica	ECM	454.52
	Juniperus deppeana	AM	274.46
	Quercus emoryi	ECM	257.59
	Juniperus monosperma	AM	241.34
	Juniperus coahuilensis	AM	228.73
	Quercus hypoleucoides	ECM	140.96
	Juniperus osteosperma	AM	4225.85
	Juniperus occidentalis	AM	1245.87

	Pseudotsuga menziesii	ECM	690.89
	Pinus ponderosa	ECM	334.29
Intermountain Semi-Desert	*Abies lasiocarpa*	ECM	312.22
	Populus tremuloides	ECM	272.52
	Juniperus scopulorum	AM	264.81
	Cercocarpus ledifolius	ECM	196.03
	Populus angustifolia	ECM	133.39
	Pinus contorta	ECM	99.72

Supplementary Table S5 | Summary of ANOVA results for forest productivity (log-transformed) when considering physiographic class. The explanatory variables include ecoregions, AM proportion (linear and quadratic terms), tree species richness (log-transformed), interactions between AM proportion and richness, interactions between AM proportion and ecoregions, stand age, elevation, slope, physiographic class, climatic variables (mean annual temperature, mean annual precipitation and temperature seasonality), and soil pH. Overall analysis (Model A) followed by separate analyses for low-richness (five or less tree species; Model B) and high-richness (more than five species; Model C) plots. Key to abbreviations: df, degree of freedom; %SS, percentage of the total sum of squares explained by each term; *F*, variance ration; *P*, probability of Type I error.

	Forest productivity				
Source of variation	df	Mean square	%SS	*F*	*P*
Model A: overall model					
Ecoregion	34	1940	46.19	2775.62	<0.001
Linear AM proportion (AM)	1	2231	1.56	3192.04	<0.001
Quadratic AM proportion (AM2)	1	2283	1.60	3265.89	<0.001
Log richness (SR)	1	13762	9.64	19689.08	<0.001
AM × SR	1	653	0.46	934.52	<0.001
AM2 × SR	1	6	0.00	8.82	0.003
AM × ecoregion	34	40	0.96	57.40	<0.001
AM2 × ecoregion	34	17	0.40	24.33	<0.001
Stand age	1	2708	1.90	3874.85	<0.001
Elevation	1	142	0.10	203.61	<0.001
Slope	1	15	0.01	21.37	<0.001
Physiographic class	1	255	0.18	364.86	<0.001
Mean annual temperature	1	2	0.00	2.31	0.129
Mean annual precipitation	1	663	0.46	948.61	<0.001
Temperature seasonality	1	97	0.07	138.68	<0.001
Soil pH	1	37	0.03	52.77	<0.001
Residuals	74441	1	36.44		
Model B: low richness					
Ecoregion	34	1150.2	37.61	1078.62	<0.001
Linear AM proportion (AM)	1	2578.5	2.48	2418.13	<0.001
Quadratic AM proportion (AM2)	1	780.9	0.75	732.29	<0.001

AM × ecoregion	34	69.7	2.28	65.38	<0.001
AM2 × ecoregion	34	16.6	0.54	15.57	<0.001
Stand age	1	1356.0	1.30	1271.67	<0.001
Elevation	1	241.0	0.23	225.97	<0.001
Slope	1	22.6	0.02	21.18	<0.001
Physiographic class	1	380.9	0.37	357.24	<0.001
Mean annual temperature	1	35.3	0.03	33.13	<0.001
Mean annual precipitation	1	1266.8	1.18	1350.50	<0.001
Temperature seasonality	1	87.0	0.08	81.58	<0.001
Soil pH	1	146.1	0.14	137.03	<0.001
Residuals	51664	1.1	52.98		
Model C: high richness					
Ecoregion	30	43.7	16.51	172.87	<0.001
Linear AM proportion (AM)	1	1.7	0.03	6.68	0.010
Quadratic AM proportion (AM2)	1	0.1	0.00	0.52	0.470
AM × ecoregion	28	2.1	0.74	8.41	<0.001
AM2 × ecoregion	28	1.1	0.38	4.26	<0.001
Stand age	1	705.2	8.89	2792.60	<0.001
Elevation	1	8.9	0.11	35.26	<0.001
Slope	1	0.0	0.00	0.01	0.942
Physiographic class	1	34.0	0.43	134.71	<0.001
Mean annual temperature	1	4.6	0.06	18.09	<0.001
Mean annual precipitation	1	10.2	0.13	40.31	<0.001
Temperature seasonality	1	36.8	0.47	145.53	<0.001
Soil pH	1	4.1	0.05	16.39	<0.001
Residuals	22689	0.3	72.21		

Supplementary Table S6 | Summary of ANOVA results for forest productivity (log-transformed) when using Shannon diversity instead of tree species richness. The explanatory variables include ecoregions, AM proportion (linear and quadratic terms), Shannon diversity (the exponential of Shannon's entropy index $q = 1$), interactions between AM proportion and diversity, interactions between AM proportion and ecoregion, stand age, elevation, slope, climatic variables (mean annual temperature, mean annual precipitation and temperature seasonality), and soil pH. Overall analysis (Model A) followed by separate analyses for low- and high-diversity plots, which did not include diversity and its interaction terms in the models. Different cut-offs of diversity were used for Model B1 & B2, Model C1&C2, and Model D1&D2. Key to abbreviations: df, degree of freedom; *F*, variance ration; *P*, probability of Type I error.

	Forest productivity			
Source of variation	df	Mean square	*F*	*P*
Model A: overall model				
Ecoregion	34	1940	2404.26	<0.001
Linear AM proportion (AM)	1	2231	2764.96	<0.001
Quadratic AM proportion (AM2)	1	2283	2828.93	<0.001
Shannon diversity (DIV)	1	4037	5003.57	<0.001
AM × DIV	1	417	516.88	<0.001
AM2 × DIV	1	1266	1569.26	<0.001
AM: ecoregion	34	59	73.58	<0.001
AM2: ecoregion	34	25	30.81	<0.001
Stand age	1	2125	2633.73	<0.001
Elevation	1	167	206.47	<0.001
Slope	1	1	1.65	<0.001
Mean annual temperature	1	15	18.92	0.199
Mean annual precipitation	1	1141	1414.32	<0.001
Temperature seasonality	1	117	145.17	<0.001
Soil pH	1	93	114.90	<0.001
Residuals	74448	1		
Model B1: diversity ≤ 3				
Ecoregion	34	952.7	815.45	<0.001
Linear AM proportion (AM)	1	2534.4	2169.23	<0.001
Quadratic AM proportion (AM2)	1	514.9	440.70	<0.001
AM × ecoregion	34	62.8	53.75	<0.001

AM2 × ecoregion	34	13.0	11.13	<0.001
Stand age	1	1109.6	949.68	<0.001
Elevation	1	222.7	190.59	<0.001
Slope	1	9.9	8.47	0.004
Mean annual temperature	1	86.9	74.35	<0.001
Mean annual precipitation	1	1270.3	1087.32	<0.001
Temperature seasonality	1	56.8	48.62	<0.001
Soil pH	1	160.7	137.53	<0.001
Residuals	42310	1.2		
Model B2: diversity >3				
Ecoregion	34	113.3	311.26	<0.001
Linear AM proportion (AM)	1	29.5	80.91	<0.001
Quadratic AM proportion (AM2)	1	23.2	63.69	<0.001
AM × ecoregion	32	6.9	18.82	<0.001
AM2 × ecoregion	32	1.9	5.17	<0.001
Stand age	1	963.4	2645.62	<0.001
Elevation	1	1.7	4.77	0.029
Slope	1	8.3	22.91	<0.001
Mean annual temperature	1	17.5	47.92	<0.001
Mean annual precipitation	1	61.8	169.64	<0.001
Temperature seasonality	1	60.4	165.18	<0.001
Soil pH	1	0.6	1.66	0.198
Residuals	32034	0.4		
Model C1: diversity ≤ 4				
Ecoregion	34	1258.2	1173.21	<0.001
Linear AM proportion (AM)	1	2491.2	2322.80	<0.001
Quadratic AM proportion (AM2)	1	988.6	921.76	<0.001
AM × ecoregion	34	71.6	66.74	<0.001
AM2 × ecoregion	34	16.9	15.80	<0.001
Stand age	1	1362.5	1270.44	<0.001
Elevation	1	251.0	234.00	<0.001
Slope	1	31.1	29.04	<0.001
Mean annual temperature	1	53.4	49.78	<0.001
Mean annual precipitation	1	1422.9	1326.77	<0.001
Temperature seasonality	1	79.5	74.10	<0.001
Soil pH	1	169.5	158.08	<0.001
Residuals	52505	1.1		
Model C2: diversity > 4				
Ecoregion	31	47.1	158.33	<0.001
Linear AM proportion (AM)	1	6.1	20.58	<0.001

Quadratic AM proportion (AM2)	1	10.7	36.07	<0.001
AM × ecoregion	29	2.4	8.13	<0.001
AM2 × ecoregion	28	1.3	4.44	<0.001
Stand age	1	535.9	1802.36	<0.001
Elevation	1	12.9	43.49	<0.001
Slope	1	0.2	0.62	0.429
Mean annual temperature	1	7.2	24.18	<0.001
Mean annual precipitation	1	11.2	37.63	<0.001
Temperature seasonality	1	37.8	127.04	<0.001
Soil pH	1	3.3	11.21	0.080
Residuals	21848	0.3		
Model D1: diversity ≤ 5				
Ecoregion	34	1491.8	1489.71	<0.001
Linear AM proportion (AM)	1	2396.5	2393.05	<0.001
Quadratic AM proportion (AM2)	1	1390.4	1388.41	<0.001
AM × ecoregion	34	74.4	74.26	<0.001
AM2 × ecoregion	34	18.5	18.46	<0.001
Stand age	1	1453.3	1451.25	<0.001
Elevation	1	235.8	235.47	<0.001
Slope	1	49.5	49.39	<0.001
Mean annual temperature	1	31.8	31.79	<0.001
Mean annual precipitation	1	1432.1	1430.06	<0.001
Temperature seasonality	1	90.6	90.46	<0.001
Soil pH	1	161.1	160.90	<0.001
Residuals	60217	1.0		
Model D2: diversity > 5				
Ecoregion	28	22.0	86.68	<0.001
Linear AM proportion (AM)	1	1.5	5.87	0.015
Quadratic AM proportion (AM2)	1	5.5	21.72	<0.001
AM × ecoregion	24	0.9	3.74	<0.001
AM2 × ecoregion	20	1.1	4.37	<0.001
Stand age	1	376.2	1483.87	<0.001
Elevation	1	7.8	30.75	<0.001
Slope	1	0.1	0.27	0.603
Mean annual temperature	1	1.7	6.66	0.010
Mean annual precipitation	1	9.4	37.15	<0.001
Temperature seasonality	1	27.5	108.30	<0.001
Soil pH	1	3.6	14.34	<0.001
Residuals	14152	0.3		

Supplementary Table S7 | Summary of ANOVA results for forest productivity (log-transformed) when using Simpson diversity instead of tree species richness. The explanatory variables include ecoregions, AM proportion (linear and quadratic terms), Simpson diversity (the inverse of Simpson's concentration index $q = 2$), interactions between AM proportion and diversity, interactions between AM proportion and ecoregions, stand age, elevation, slope, climatic variables (mean annual temperature, mean annual precipitation and temperature seasonality), and soil pH. Overall analysis (Model A) followed by separate analyses for low- and high-diversity plots, which did not include diversity and its interaction terms in the models. Different cut-offs of diversity were used for Model B1 & B2, Model C1&C2, and Model D1&D2. Key to abbreviations: df, degree of freedom; *F*, variance ration; *P*, probability of Type I error.

	Forest productivity			
Source of variation	df	Mean square	*F*	*P*
Model A: overall model				
Ecoregion	34	1940.0	2335.33	<0.001
Linear AM proportion (AM)	1	2231.1	2685.69	<0.001
Quadratic AM proportion (AM2)	1	2282.7	2747.83	<0.001
Simpson diversity (DIV)	1	2425.2	2919.40	<0.001
AM × DIV	1	307.1	369.70	<0.001
AM2 × DIV	1	1094.6	1317.60	<0.001
AM × ecoregion	34	62.6	75.37	<0.001
AM2 × ecoregion	34	26.0	31.26	<0.001
Stand age	1	1992.7	2398.76	<0.001
Elevation	1	177.4	213.54	<0.001
Slope	1	11.6	13.98	<0.001
Mean annual temperature	1	12.5	15.04	<0.001
Mean annual precipitation	1	1219.9	1468.46	<0.001
Temperature seasonality	1	111.5	134.21	<0.001
Soil pH	1	106.7	128.49	<0.001
Residuals	74448	0.8		
Model B1: diversity ≤ 3				
Ecoregion	34	1205.1	1094.39	<0.001
Linear AM proportion (AM)	1	2456.7	2230.99	<0.001
Quadratic AM proportion (AM2)	1	980.0	889.94	<0.001
AM × ecoregion	34	68.3	61.99	<0.001

AM2 × ecoregion	34	15.8	14.37	<0.001
Stand age	1	1239.5	1125.65	<0.001
Elevation	1	240.5	218.39	<0.001
Slope	1	31.0	28.17	<0.001
Mean annual temperature	1	57.7	52.43	<0.001
Mean annual precipitation	1	1353.6	1229.24	<0.001
Temperature seasonality	1	69.7	63.26	<0.001
Soil pH	1	181.6	164.90	<0.001
Residuals	49007	1.1		
Model B2: diversity >3				
Ecoregion	33	80.3	226.90	<0.001
Linear AM proportion (AM)	1	22.6	63.91	<0.001
Quadratic AM proportion (AM2)	1	30.4	85.90	<0.001
AM × ecoregion	31	4.5	12.78	<0.001
AM2 × ecoregion	29	2.1	5.96	<0.001
Stand age	1	668.1	1888.26	<0.001
Elevation	1	5.0	14.04	<0.001
Slope	1	2.8	7.97	0.005
Mean annual temperature	1	10.1	28.51	<0.001
Mean annual precipitation	1	41.2	116.39	<0.001
Temperature seasonality	1	42.8	118.18	<0.001
Soil pH	1	2.3	6.59	0.010
Residuals	25341	0.4		
Model C1: diversity ≤ 4				
Ecoregion	34	1499.5	1490.95	<0.001
Linear AM proportion (AM)	1	2374.0	2360.47	<0.001
Quadratic AM proportion (AM2)	1	1485.5	1477.11	<0.001
AM × ecoregion	34	73.5	73.13	<0.001
AM2 × ecoregion	34	18.7	18.61	<0.001
Stand age	1	1427.5	1419.42	<0.001
Elevation	1	240.9	239.56	<0.001
Slope	1	54.7	54.43	<0.001
Mean annual temperature	1	32.5	32.35	<0.001
Mean annual precipitation	1	1432.1	1423.95	<0.001
Temperature seasonality	1	84.4	83.90	<0.001
Soil pH	1	166.5	165.56	<0.001
Residuals	59317	1.0		
Model C2: diversity > 4				
Ecoregion	30	28.0	94.88	<0.001
Linear AM proportion (AM)	1	6.0	20.34	<0.001

Quadratic AM proportion (AM2)	1	10.2	34.56	<0.001
AM × ecoregion	26	1.8	6.01	<0.001
AM2 × ecoregion	25	1.2	3.98	<0.001
Stand age	1	369.2	1250.89	<0.001
Elevation	1	10.0	33.91	<0.001
Slope	1	0.0	0.05	0.822
Mean annual temperature	1	3.2	10.93	<0.001
Mean annual precipitation	1	10.8	36.49	<0.001
Temperature seasonality	1	31.8	107.86	<0.001
Soil pH	1	3.7	12.43	<0.001
Residuals	15043	0.3		
Model D1: diversity ≤ 5				
Ecoregion	34	1692.0	1783.93	<0.001
Linear AM proportion (AM)	1	2295.6	2420.32	<0.001
Quadratic AM proportion (AM2)	1	1807.9	1906.08	<0.001
AM × ecoregion	34	75.0	79.05	<0.001
AM2 × ecoregion	34	19.5	20.15	<0.001
Stand age	1	1495.6	1576.84	<0.001
Elevation	1	223.2	235.37	<0.001
Slope	1	67.7	71.39	<0.001
Mean annual temperature	1	19.4	20.50	<0.001
Mean annual precipitation	1	1461.5	1540.85	<0.001
Temperature seasonality	1	89.4	94.22	<0.001
Soil pH	1	155.8	164.28	<0.001
Residuals	66063	0.9		
Model D2: diversity > 5				
Ecoregion	20	13.8	55.26	<0.001
Linear AM proportion (AM)	1	3.2	12.97	<0.001
Quadratic AM proportion (AM2)	1	6.8	27.10	<0.001
AM × ecoregion	16	1.1	4.26	<0.001
AM2 × ecoregion	16	1.1	4.44	<0.001
Stand age	1	231.0	925.97	<0.001
Elevation	1	3.9	15.71	<0.001
Slope	1	0.0	0.10	0.754
Mean annual temperature	1	0.5	2.09	0.148
Mean annual precipitation	1	5.9	23.56	<0.001
Temperature seasonality	1	23.2	92.97	<0.001
Soil pH	1	5.2	20.95	<0.001
Residuals	8326	0.3		

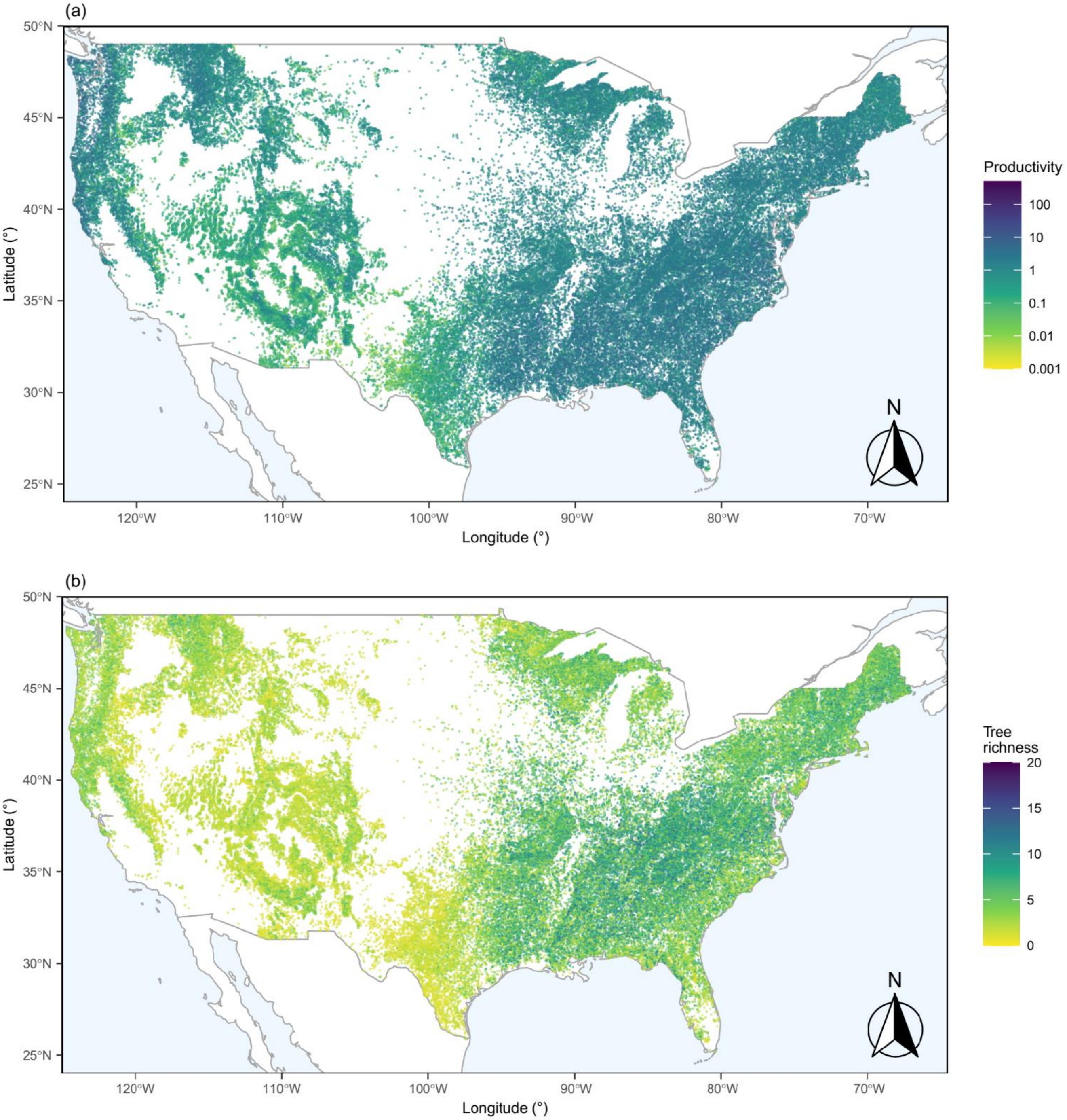
(a)
50°N
45°N
40°N
35°N
30°N
25°N
Latitude (°)
120°W
110°W
100°W
90°W
80°W
70°W
Longitude (°)
Productivity
100
10
1
0.1
0.01
0.001
N
(b)
50°N
45°N
40°N
35°N
30°N
25°N
Latitude (°)
120°W
110°W
100°W
90°W
80°W
70°W
Longitude (°)
Tree
richness
20
15
10
5
0
N

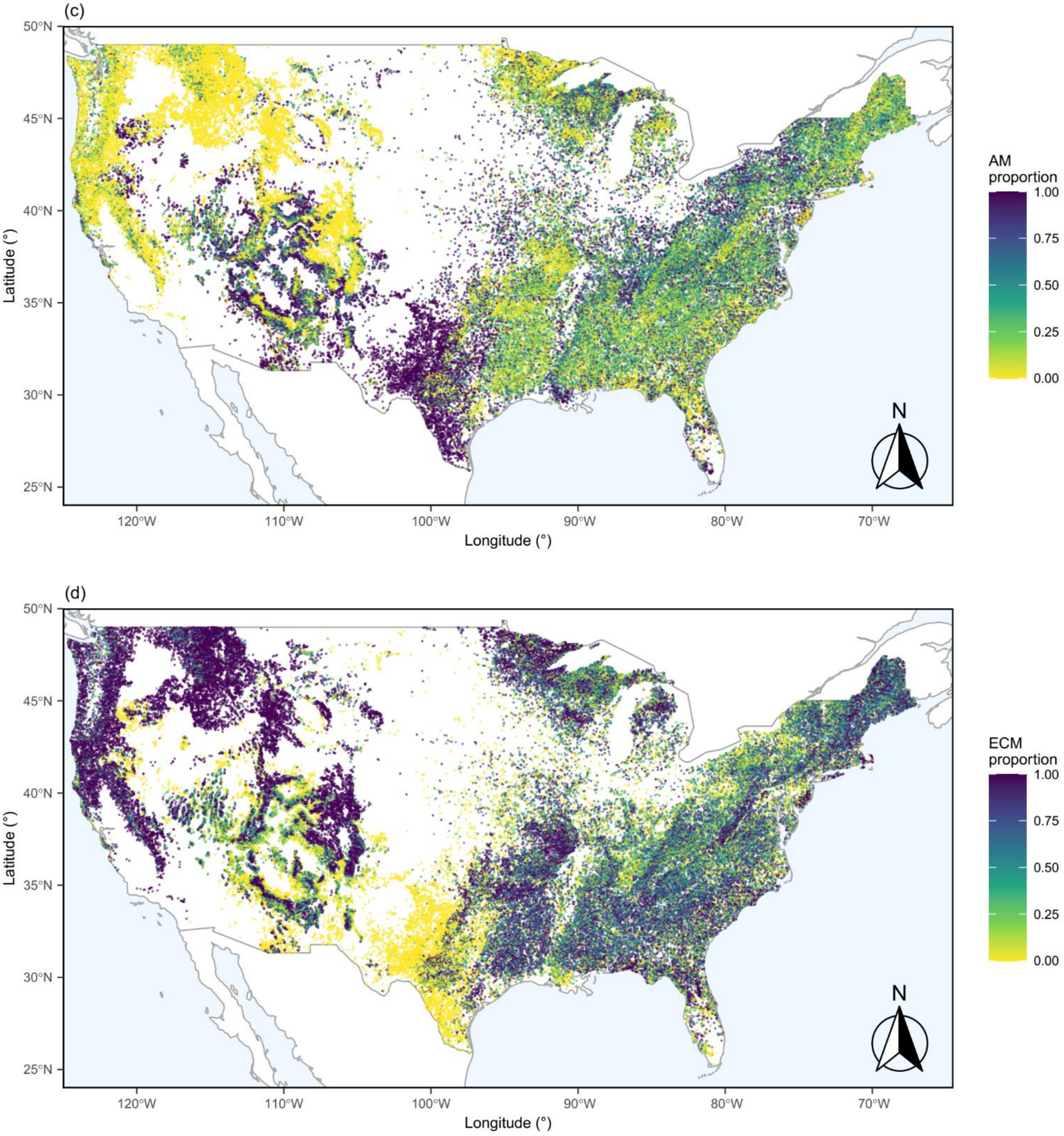

Supplementary Fig. S1 | Forest productivity, tree species richness, arbuscular mycorrhizal (AM), and ectomycorrhizal (ECM) proportions at the plot scale across the USA. a, Map of forest productivity (as total aboveground live biomass divided by stand age). b, Map of tree species

richness (the number of tree species). c, Map of AM proportion (as the proportion of basal area per plot of trees known to associate with AM fungi). d, Map of ECM proportion (as the proportion of basal area per plot of trees known to associate with ECM fungi).

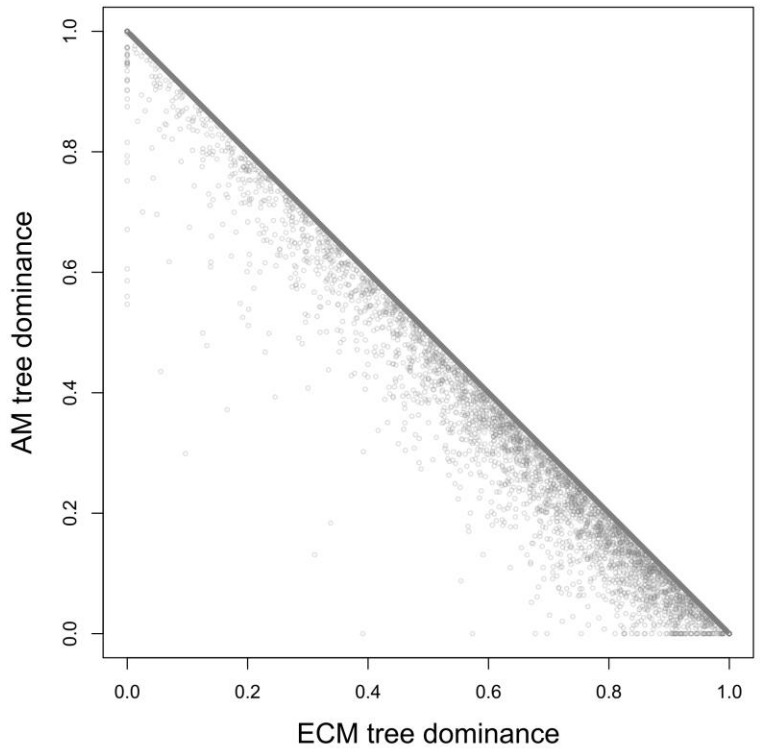

Supplementary Fig. S2 | Relationship between arbuscular mycorrhizal (AM) and ectomycorrhizal (ECM) proportions in each plot. Overall, 96.2 % of the plots have a cumulative sum of AM and ECM proportions > 0.99 (that is, most plots are located on the diagonal). Other plots (that is, below the diagonal) contain ericoid or non-mycorrhizal trees.

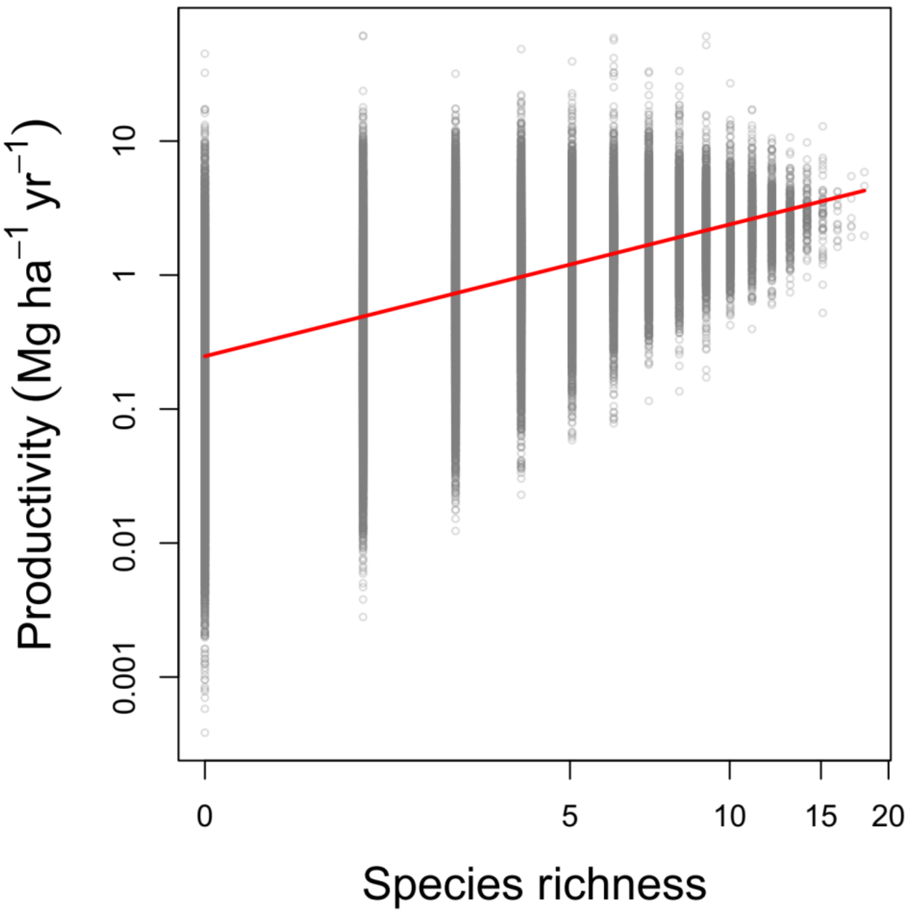

Supplementary Fig. S3 | Relationship between forest productivity and tree species richness.

See Supplementary Table S1 for statistical results.

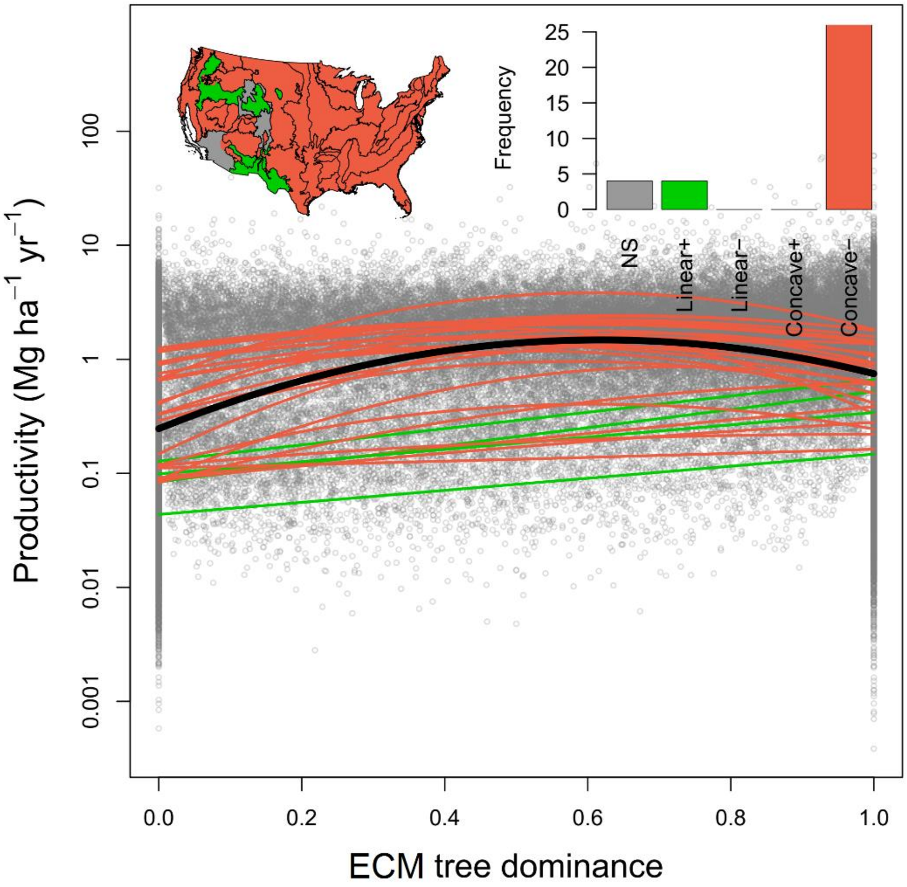

Supplementary Fig. S4 | Observed relationship between ECM tree dominance and forest productivity. ECM tree dominance is quantified as ECM proportion based on tree basal area. The black curve represents a simple regression fitted across all forest plots, whereas the other curves were simple regressions fitted for plots within each ecoregion. Each grey circle represents the data of one forest plot (n = 74,563). Inset frequency chart: The frequencies of each form of relationship observed across all ecoregions. Inset map: The colored map indicates the distribution of each form of relationship across ecoregions. The significance of the relationships in the frequency chart and colored map was based on regressions with environmental variables fitted as covariates. NS, non-significant. See Supplementary Data 2 for statistical results.

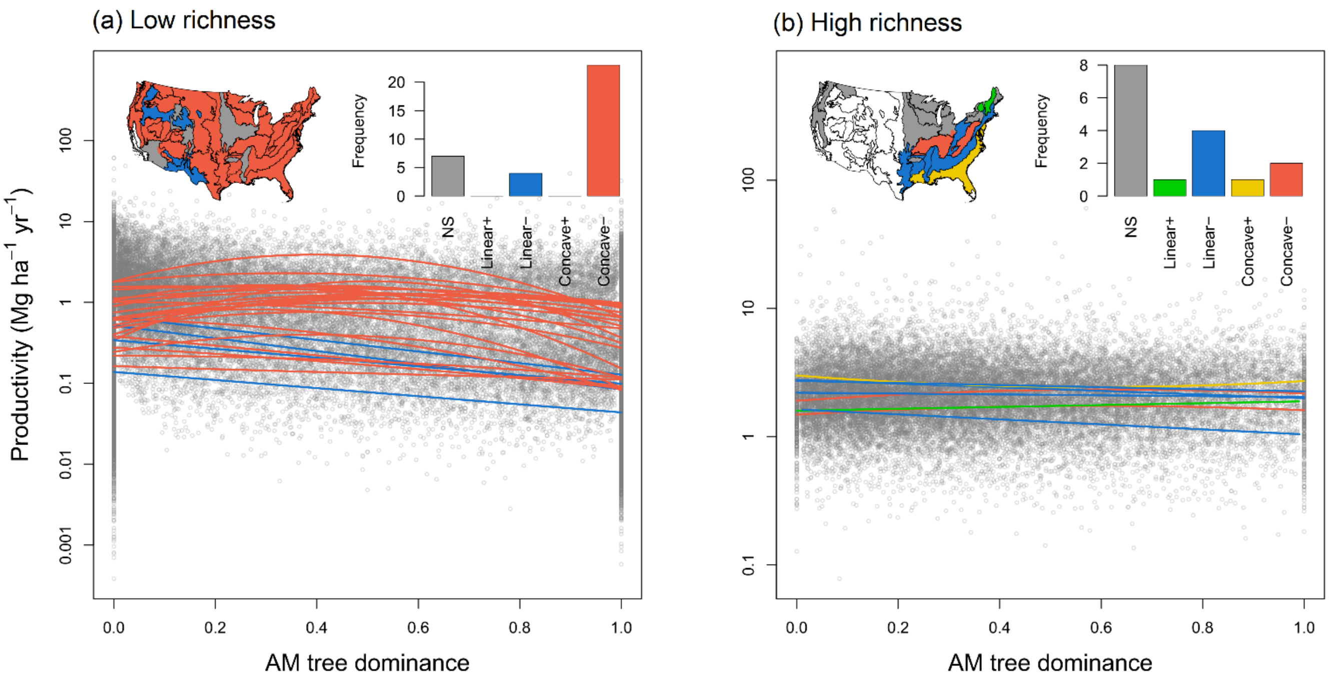

Supplementary Fig. S5 | Ecoregion-level relationships between AM tree dominance and forest productivity. a, forests with low tree species richness (richness ≤ 5); b, forests with high tree species richness (richness > 5). Colored lines indicate significant regressions for specific ecoregions. Inset frequency chart: The frequencies of each form of relationships observed across ecoregions. Inset map: The colored map indicates the distribution of each form of relationships across ecoregions. The significance of the relationships in the frequency chart and colored map was based on regressions with environmental variables fitted as covariates. NS, non-significant. The regions with white color are ecoregions that have fewer than 50 plots and were excluded from analyses. See Supplementary Data 3&4 for statistical results and Supplementary Fig. S15&16 for figures of each ecoregion.

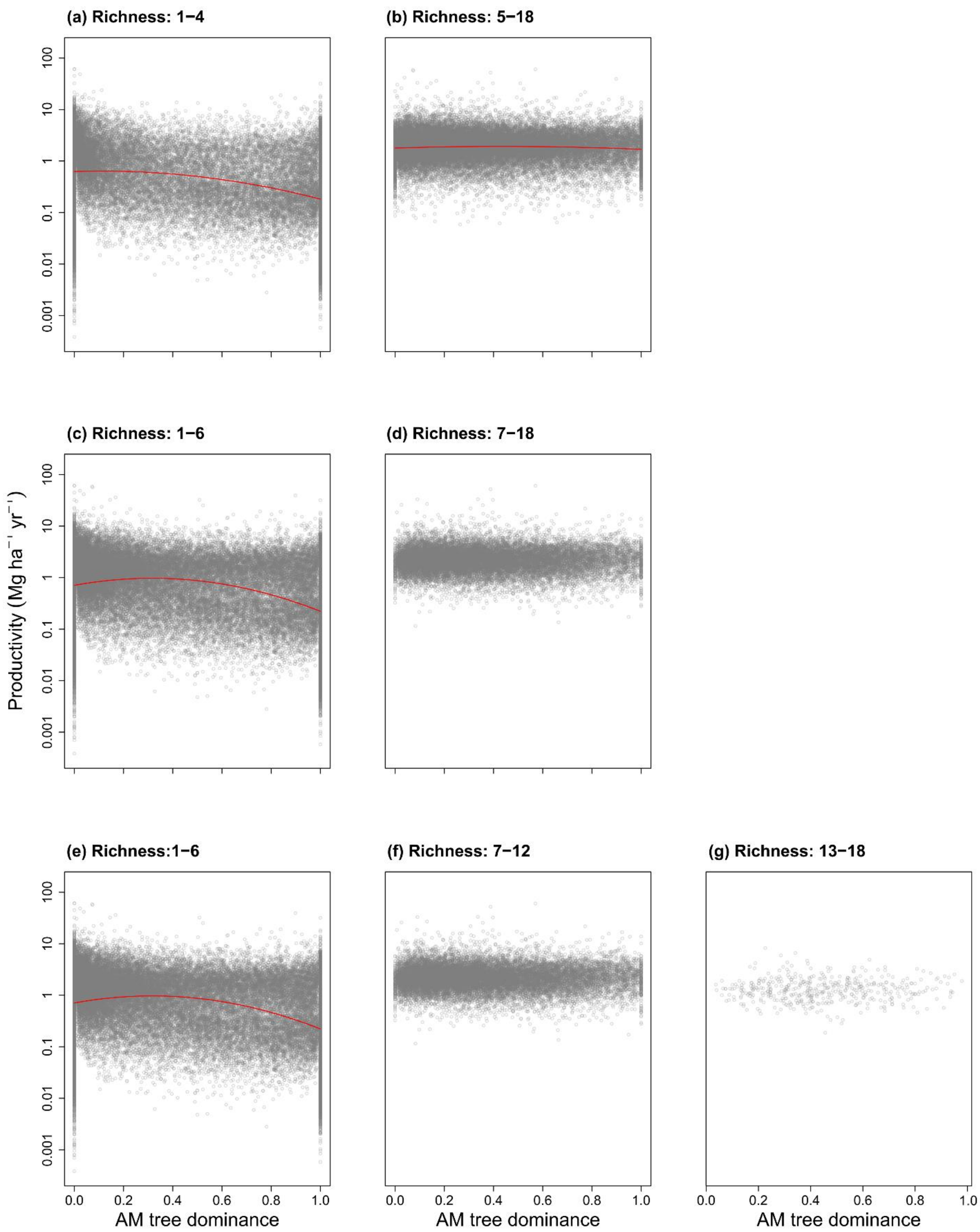

Supplementary Fig. S6 | Relationships between productivity and AM tree dominance when using different thresholds for splitting richness levels. (a-b), plots with low (richness ≤ 4) or

high species richness (richness > 4); (c-d), plots with low (richness ≤ 6) or high species richness (richness > 6); (d-g), plots with low (richness ≤ 6), intermediate (6 < richness ≤ 12) or high species richness (richness >12). We fitted general linear models with ecoregion, AM proportion (linear and quadratic terms), interactions between AM proportion and ecoregion, stand age, elevation, slope, climatic variables, and soil pH as explanatory variables (see Supplementary Table S2 for statistical results).

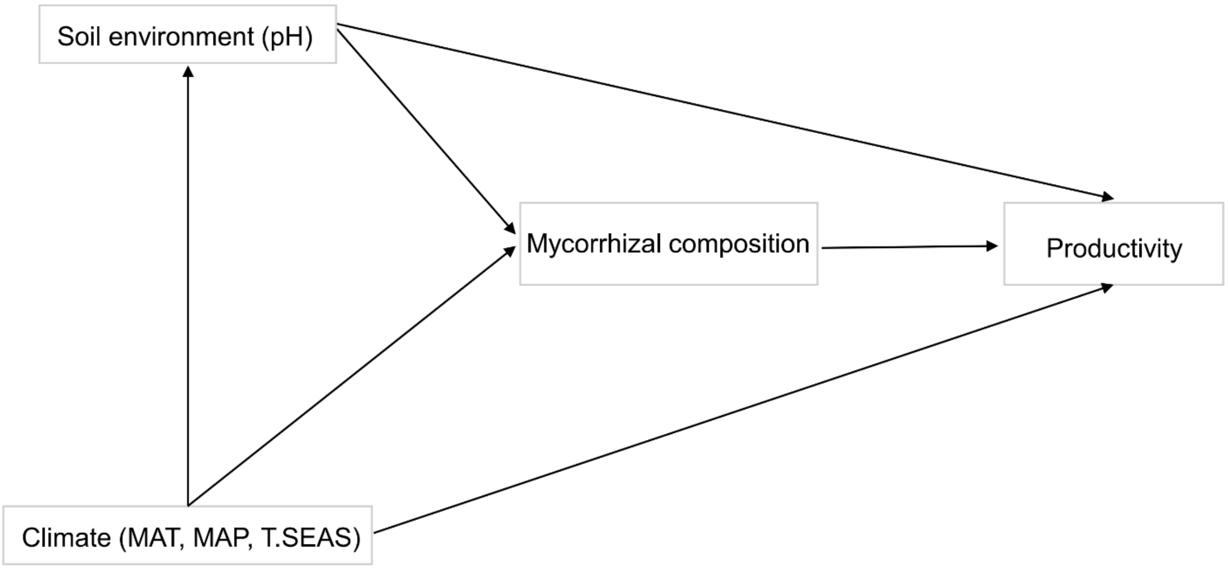

Supplementary Fig. S7 | Hypothetical diagram for structure-equation models (SEMs). The SEMs were constructed separately for low- and high-richness plots. The causal link between climate and soil environment is based on the finding of climatic controls on the release of soil nutrients from leaf litter[58], which may consequently influence soil pH[62]. MAT, mean annual temperature; MAP, mean annual precipitation; T.SEAS, temperature seasonality.

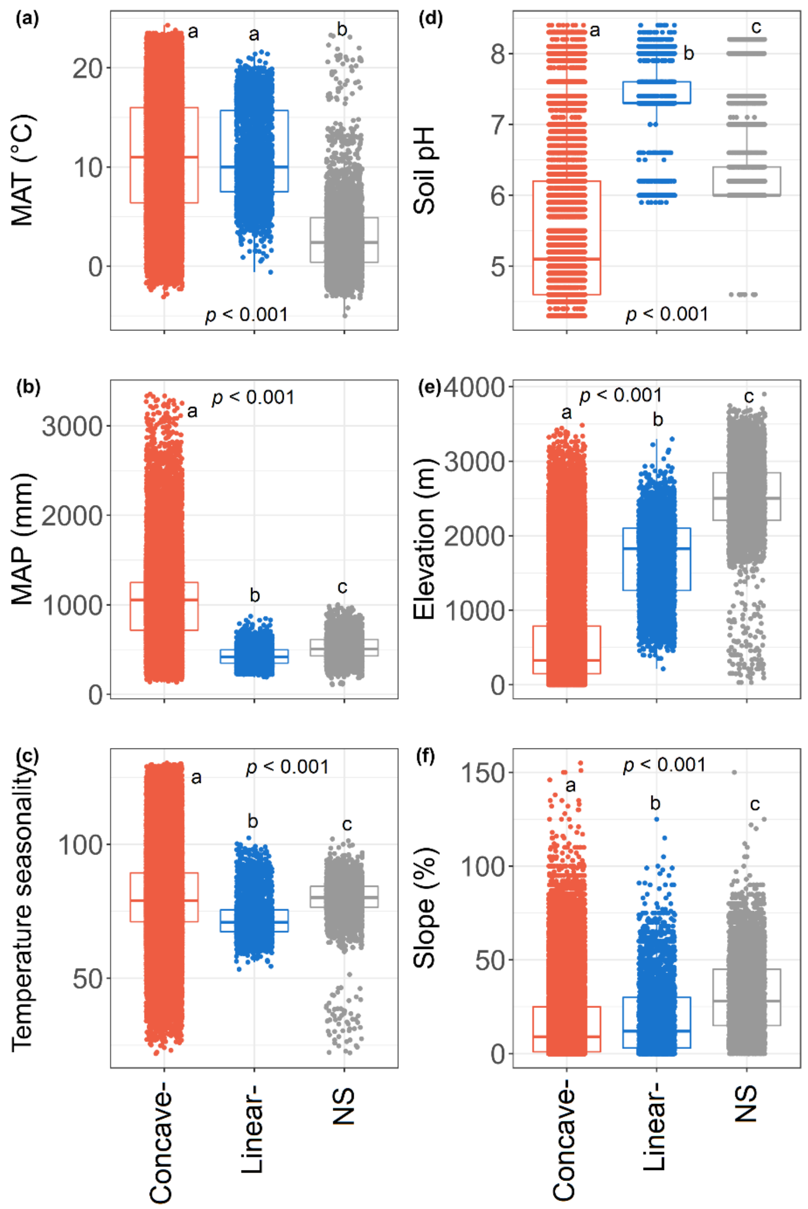

Supplementary Fig. S8 | Environmental variables grouped by ecoregions with different relationships between AM tree dominance and forest productivity. 26 ecoregions showed concave negative (concave-; n = 66,980 plots), 4 ecoregions showed linear negative (linear-; n = 3,425 plots), and the other 4 ecoregions showed non-significant (NS; n = 4,126 plots) relationships. Each point represents the data of one forest plot. Center line indicates the median value and box limits indicate upper and lower quartiles, respectively. All variables were significantly different across different groups of ecoregions ($p < 0.001$ in ANOVA). Different letters beside points

indicate significant differences (Tukey HSD) between ecoregion groups. Source data are provided as a Source Data file.

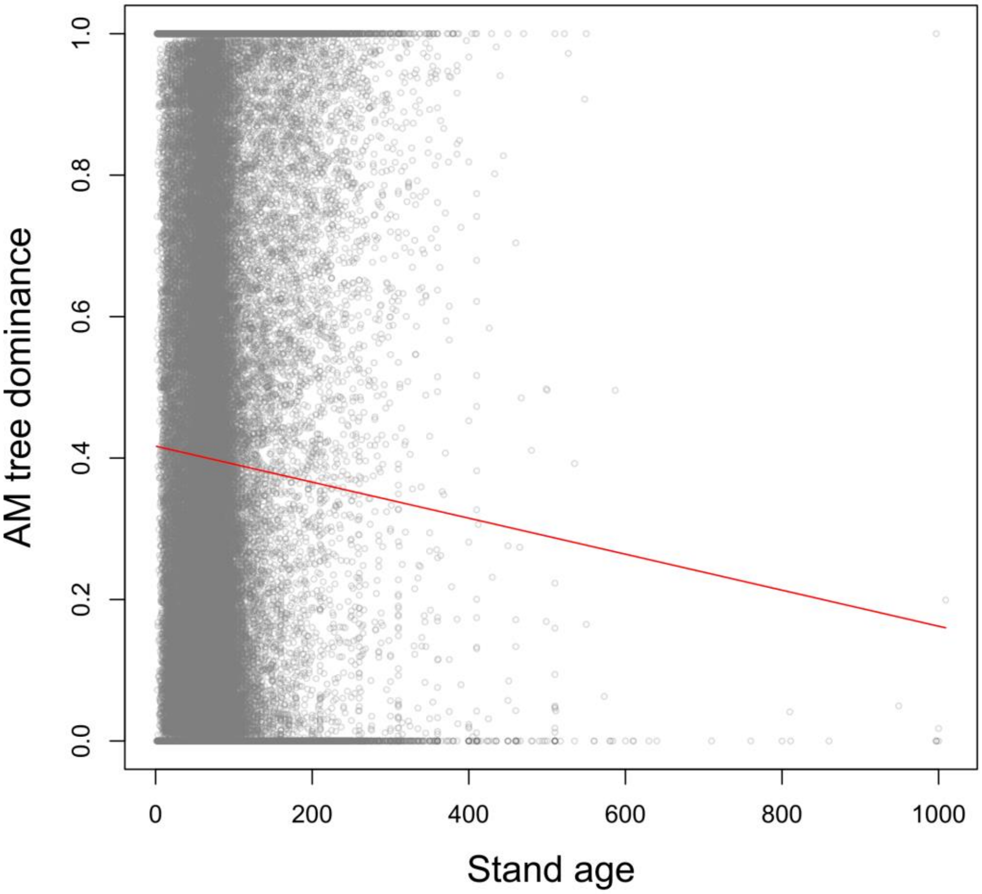

Supplementary Fig. S9 | Relationship between AM tree dominance and stand age. There is a weak but significant negative (R^2 = 0.002, $p < 0.001$ in ANOVA) relationship with AM stands on average 10.6 years younger than ECM stands.

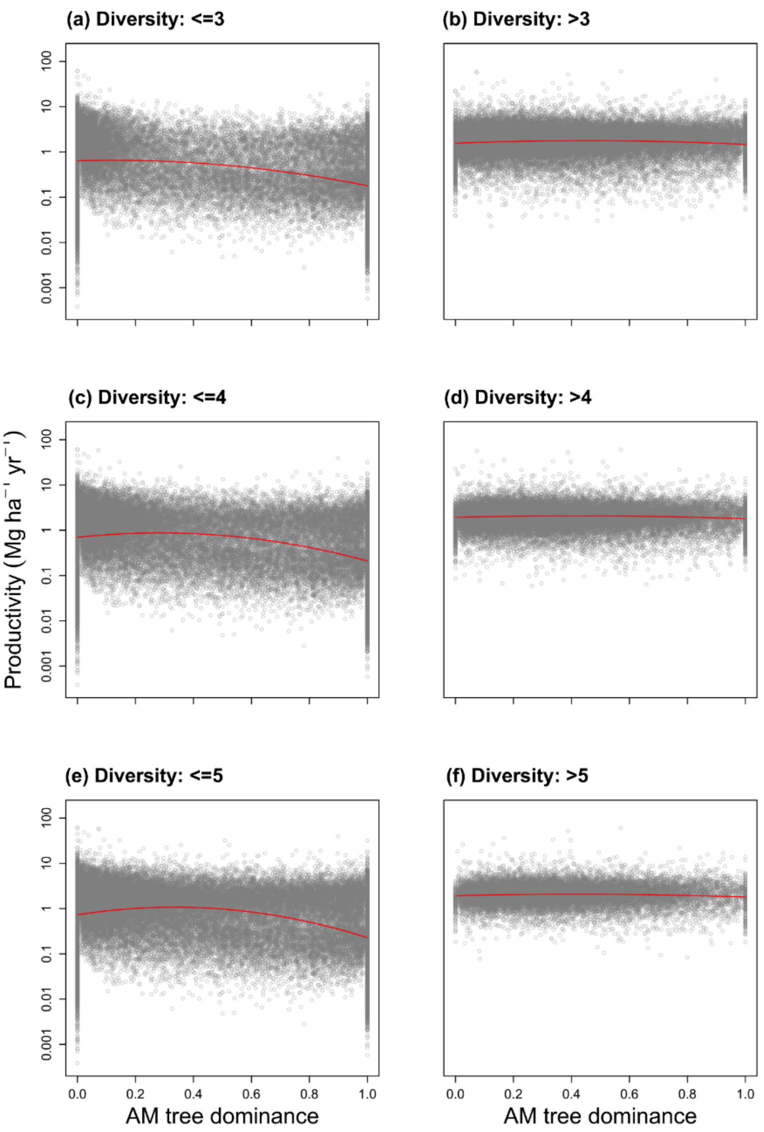

Supplementary Fig. S10 | Relationships between productivity and AM tree dominance when using different thresholds for splitting the exponential of Shannon's entropy index (q = 1). (a-b), plots with low (diversity ≤ 3) or high species diversity (diversity > 3); (c-d), plots with low (diversity ≤ 4) or high species diversity (diversity > 4); (d-g), plots with low (diversity ≤ 5), or high species diversity (diversity > 5). The mean of the exponential of Shannon's entropy index is ~2.75. We fitted general linear models with ecoregion, AM proportion (linear and quadratic terms), interactions between AM proportion and ecoregion, stand age, elevation, slope, climatic variables, and soil pH as explanatory variables (see Supplementary Table S6 for statistical results).

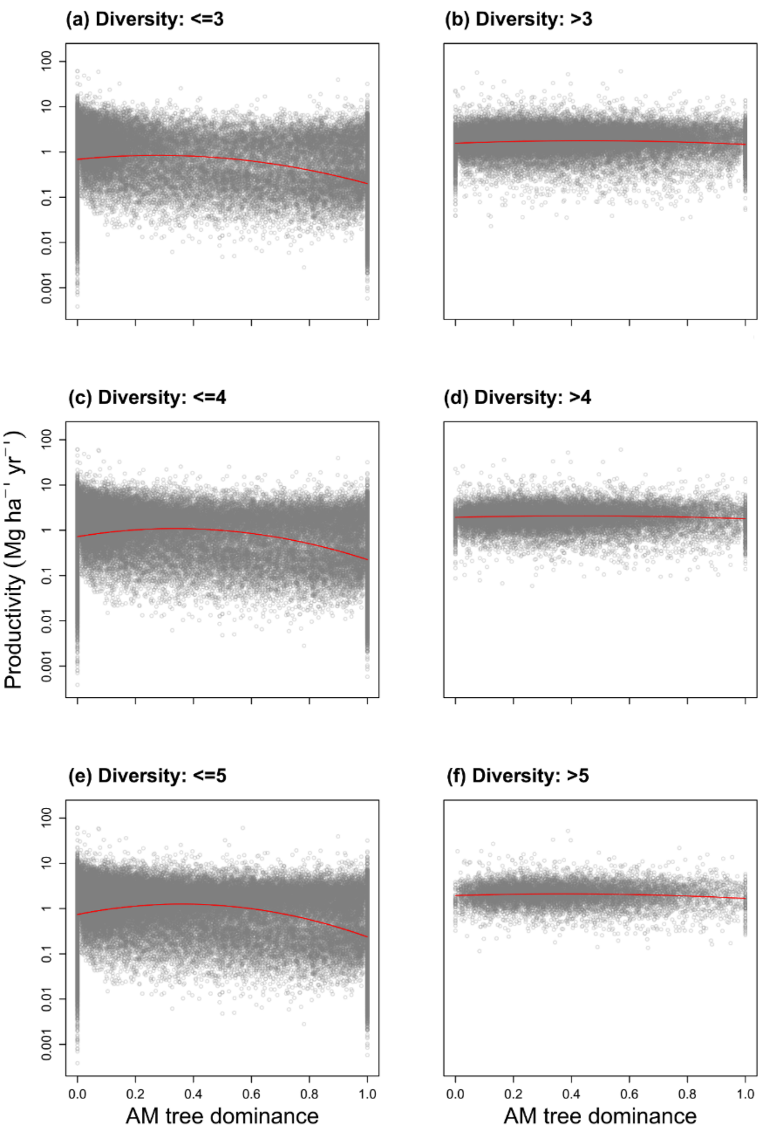

Supplementary Fig. S11 | Relationships between productivity and AM tree dominance when using different thresholds for splitting the inverse of Simpson's concentration index (*q* = 2). (a-b), plots with low (diversity ≤ 3) or high species diversity (diversity > 3); (c-d), plots with low (diversity ≤ 4) or high species diversity (diversity > 4); (d-g), plots with low (diversity ≤ 5), or high species diversity (diversity > 5). The mean of the inverse of Simpson's concentration index is ~3.23. We fitted general linear models with ecoregion, AM proportion (linear and quadratic terms), interactions between AM proportion and ecoregion, stand age, elevation, slope, climatic variables, and soil pH as explanatory variables (see Supplementary Table S7 for statistical results).

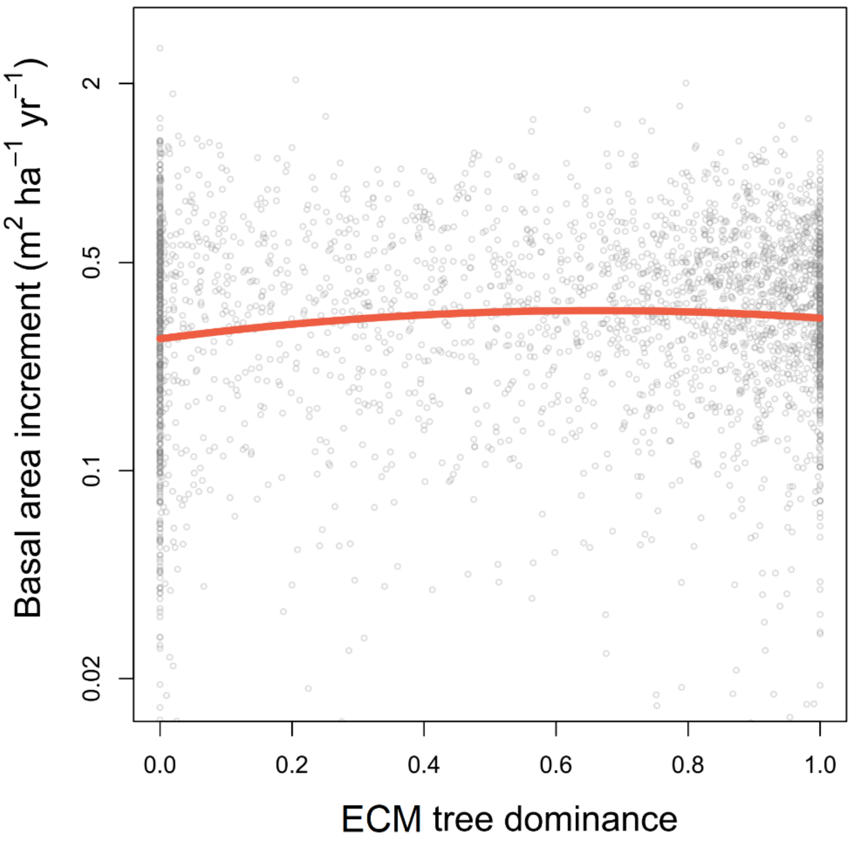

Supplementary Fig. S12 | Relationship between ECM tree dominance and basal area increment of forests in the Indiana state, USA. ECM tree dominance is quantified as ECM proportion based on tree basal area. The black curve represents a simple regression fitted across all plots, which presumably share similar environmental conditions. Each grey circle represents the data of one forest plot (n = 2,771). Basal area increment is averaged over five years (2011–2015, 2012–2016, 2013–2017 or 2014–2018). Source data are provided as a Source Data file.

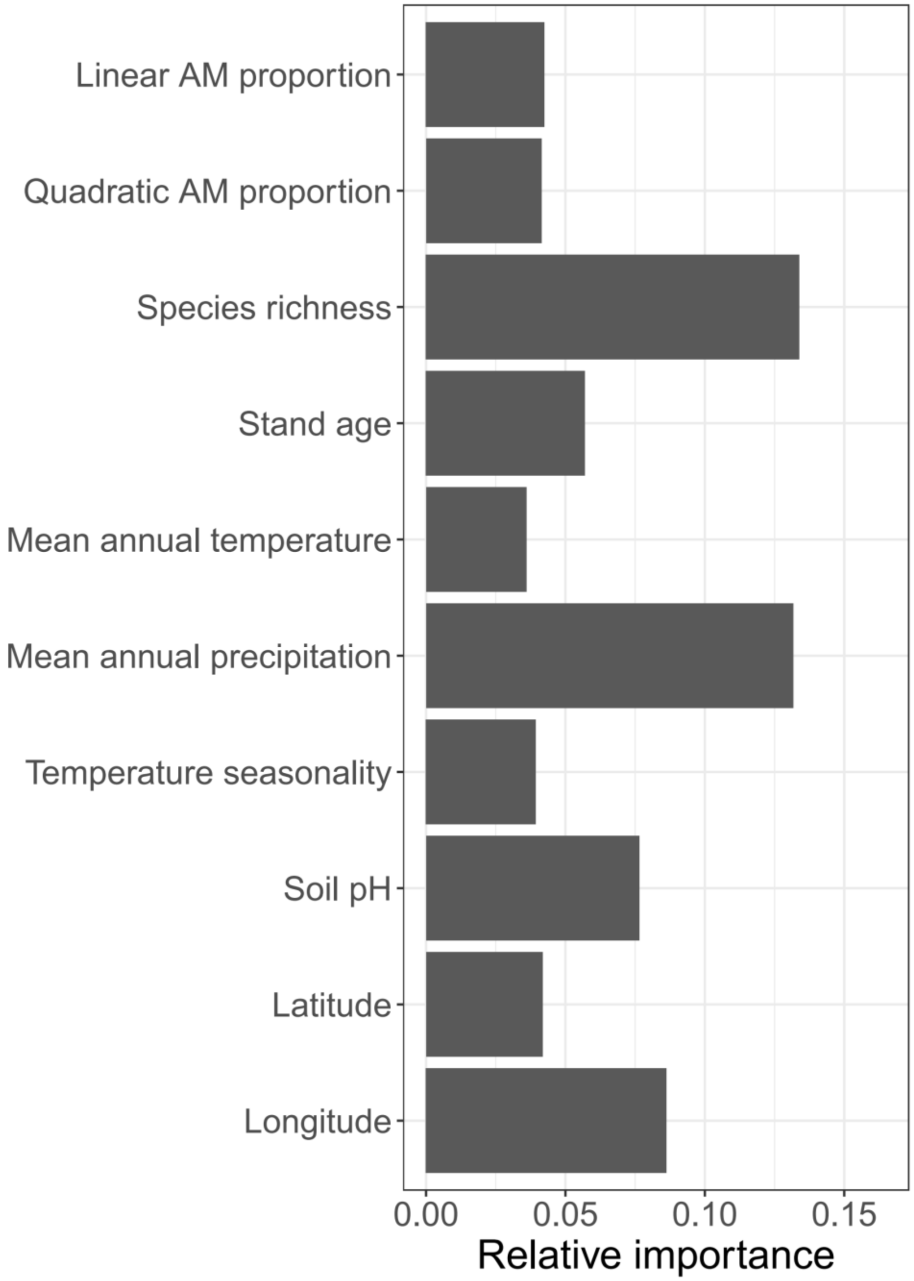

Supplementary Fig. S13 | Relative variable importance from random forest models explaining forest productivity. Relative variable importance is the mean decrease in squared error caused by each of the variables, rescaled such that it sums up to the total pseudo-R^2 of the whole model. The overall explained variation (R^2) of forest productivity (log-transformed) is 0.69. Source data are provided as a Source Data file.

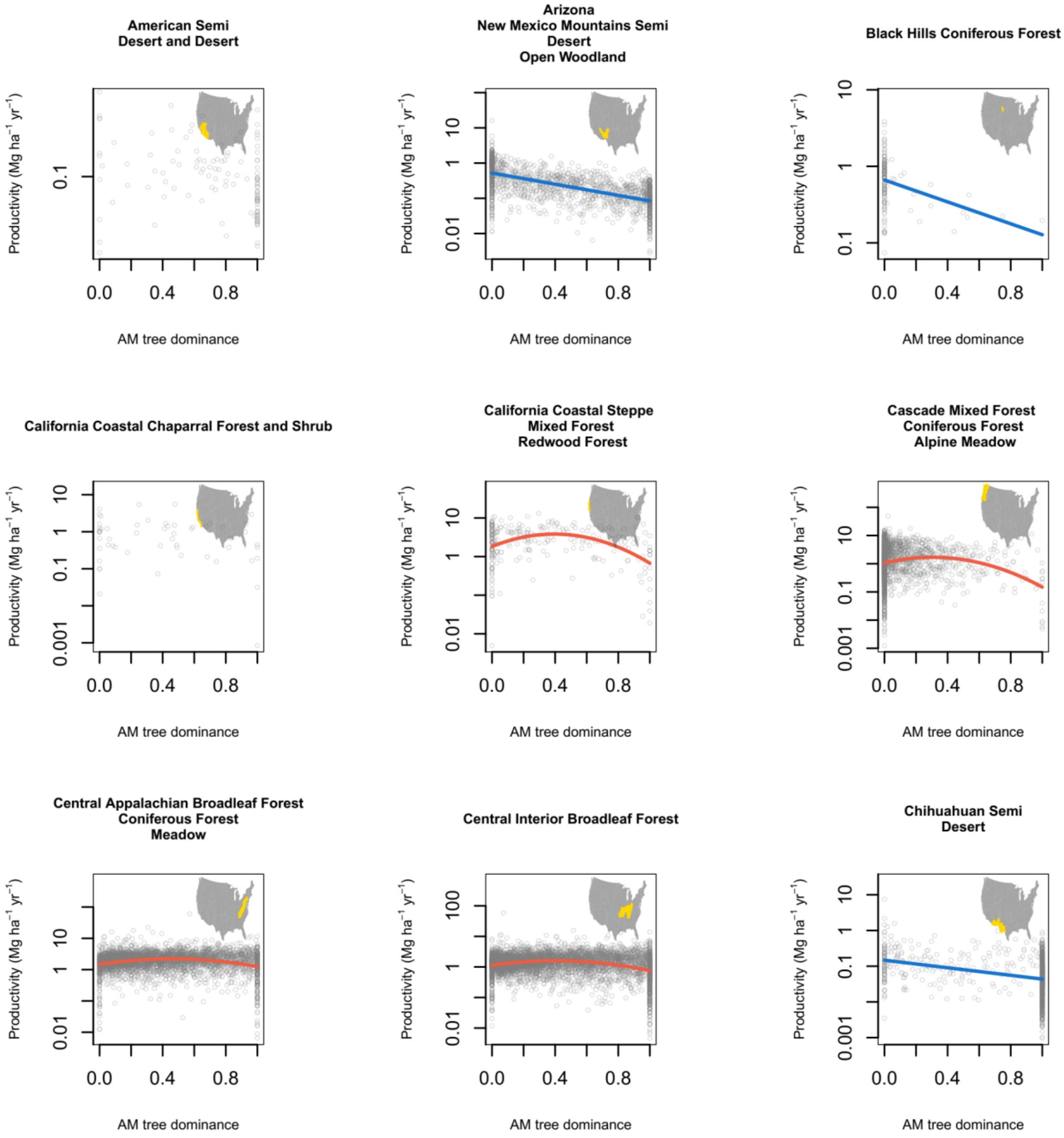
American Semi Desert and Desert
Arizona New Mexico Mountains Semi Desert Open Woodland
Black Hills Coniferous Forest
California Coastal Chaparral Forest and Shrub
California Coastal Steppe Mixed Forest Redwood Forest
Cascade Mixed Forest Coniferous Forest Alpine Meadow
Central Appalachian Broadleaf Forest Coniferous Forest Meadow
Central Interior Broadleaf Forest
Chihuahuan Semi Desert
Productivity (Mg ha^{-1} yr^{-1})
AM tree dominance
0.0
0.4
0.8
0.001
0.01
0.1
1
10
100

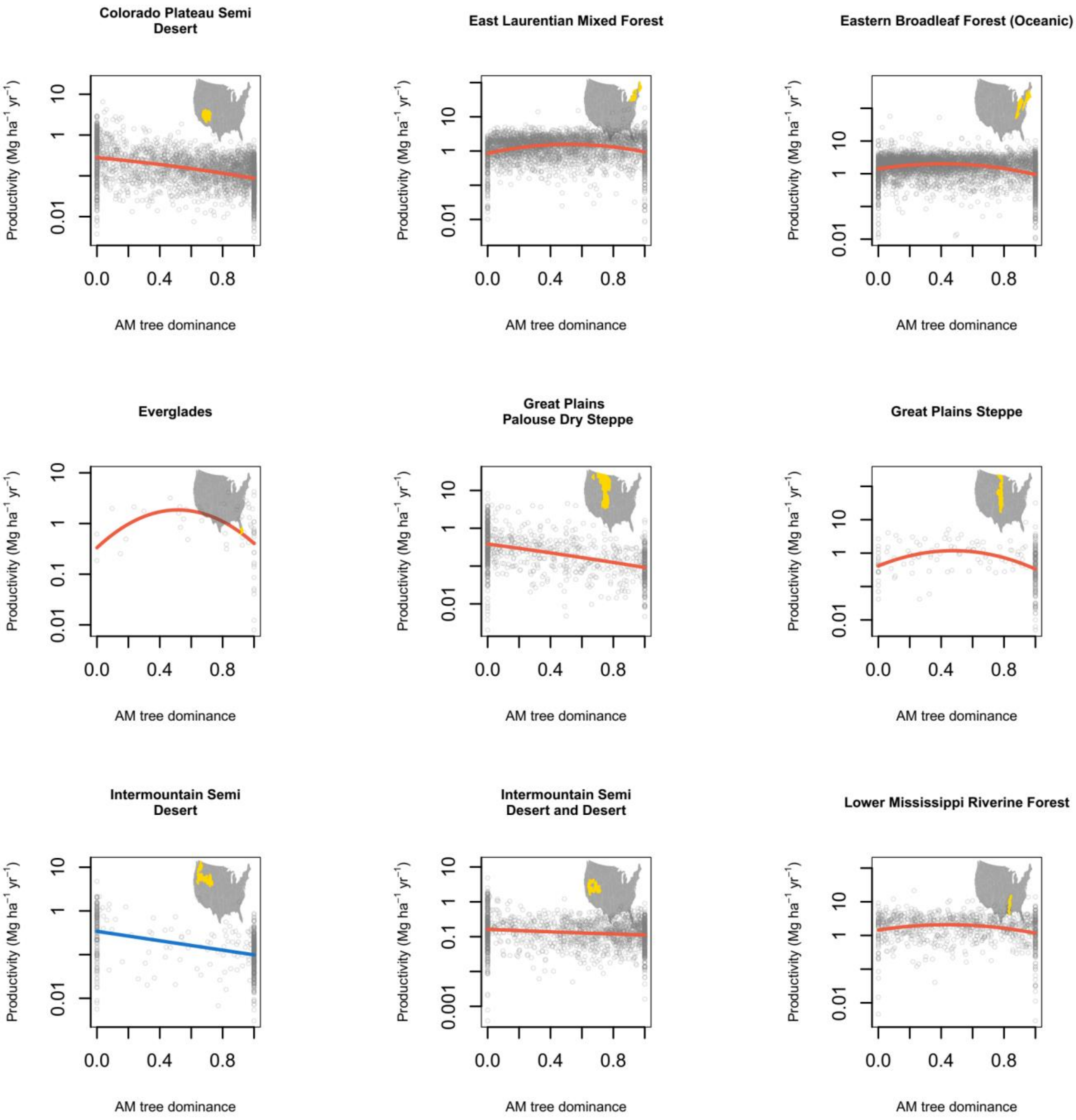
Colorado Plateau Semi Desert
East Laurentian Mixed Forest
Eastern Broadleaf Forest (Oceanic)
Everglades
Great Plains Palouse Dry Steppe
Great Plains Steppe
Intermountain Semi Desert
Intermountain Semi Desert and Desert
Lower Mississippi Riverine Forest
Productivity (Mg ha^{-1} yr^{-1})
AM tree dominance
0.0
0.4
0.8
0.001
0.01
0.1
1
10

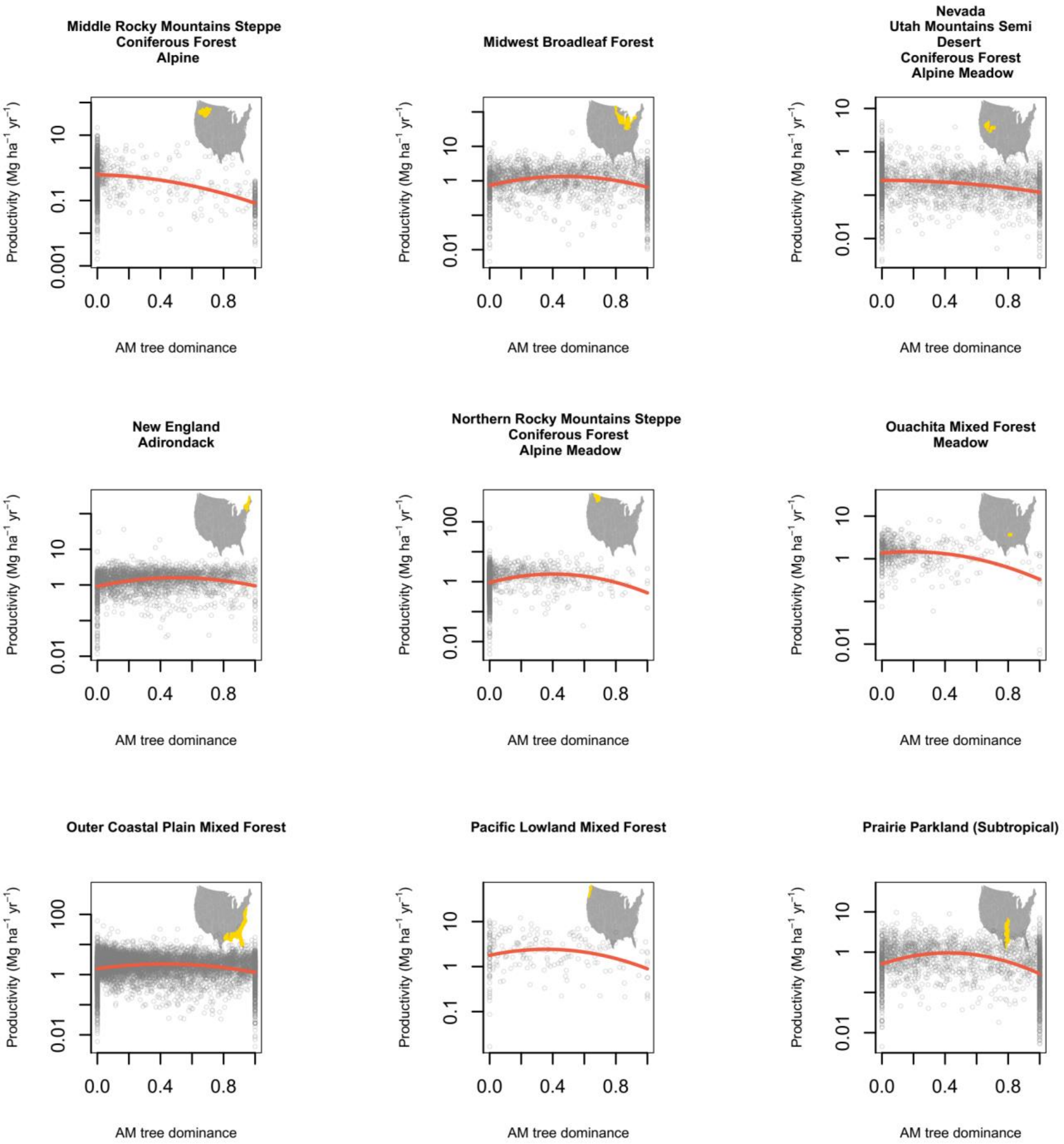

Middle Rocky Mountains Steppe
Coniferous Forest
Alpine
Midwest Broadleaf Forest
Nevada
Utah Mountains Semi
Desert
Coniferous Forest
Alpine Meadow
New England
Adirondack
Northern Rocky Mountains Steppe
Coniferous Forest
Alpine Meadow
Ouachita Mixed Forest
Meadow
Outer Coastal Plain Mixed Forest
Pacific Lowland Mixed Forest
Prairie Parkland (Subtropical)
Productivity (Mg ha^{-1} yr^{-1})
AM tree dominance
0.0
0.4
0.8
0.001
0.01
0.1
1
10
100

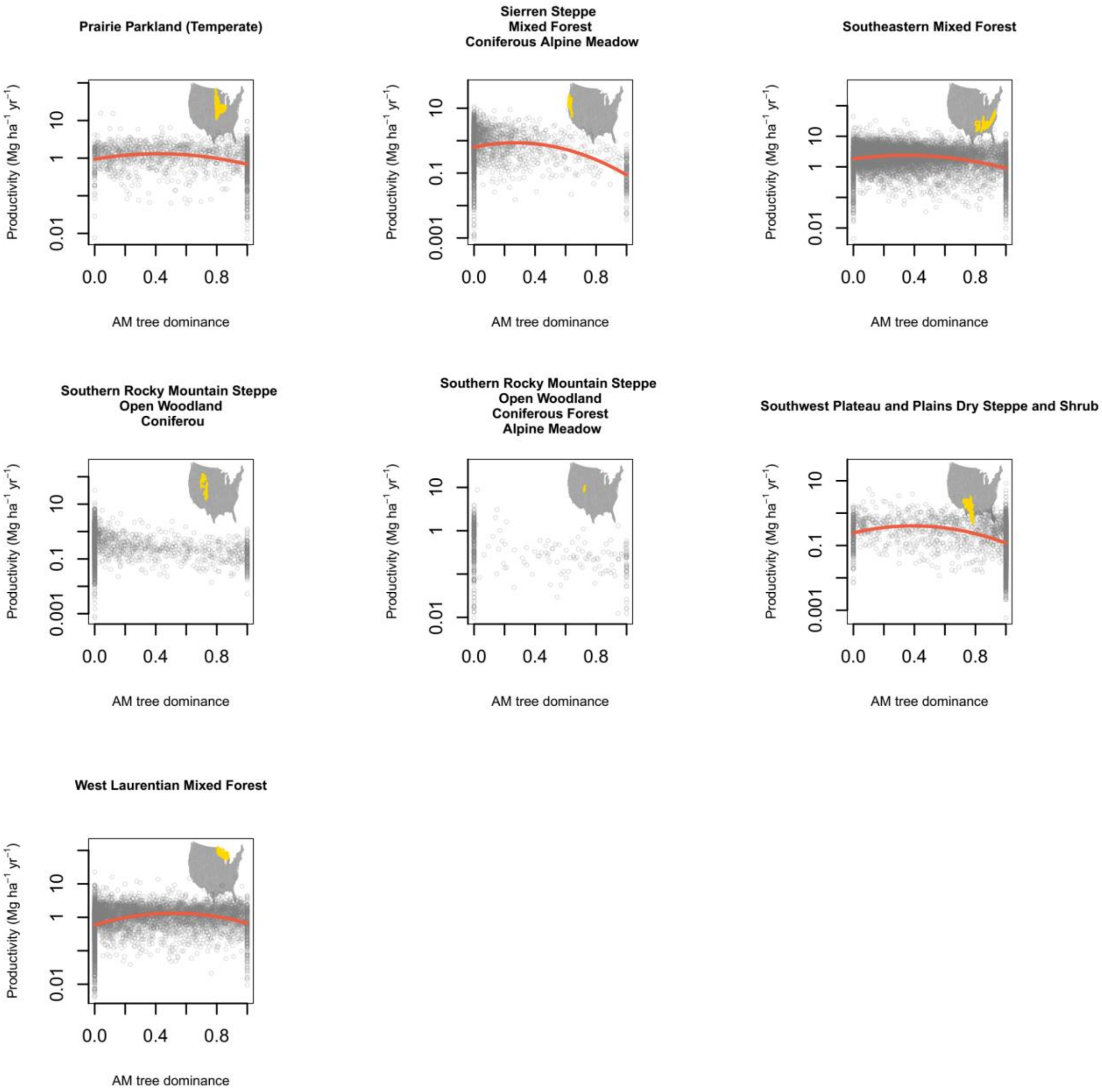

Supplementary Fig. S14 | Ecoregion-level relationships between AM tree dominance and productivity across all plots. The colored area in the inset map indicates the distribution of the corresponding ecoregion. See Supplementary Data 1 for statistical results.

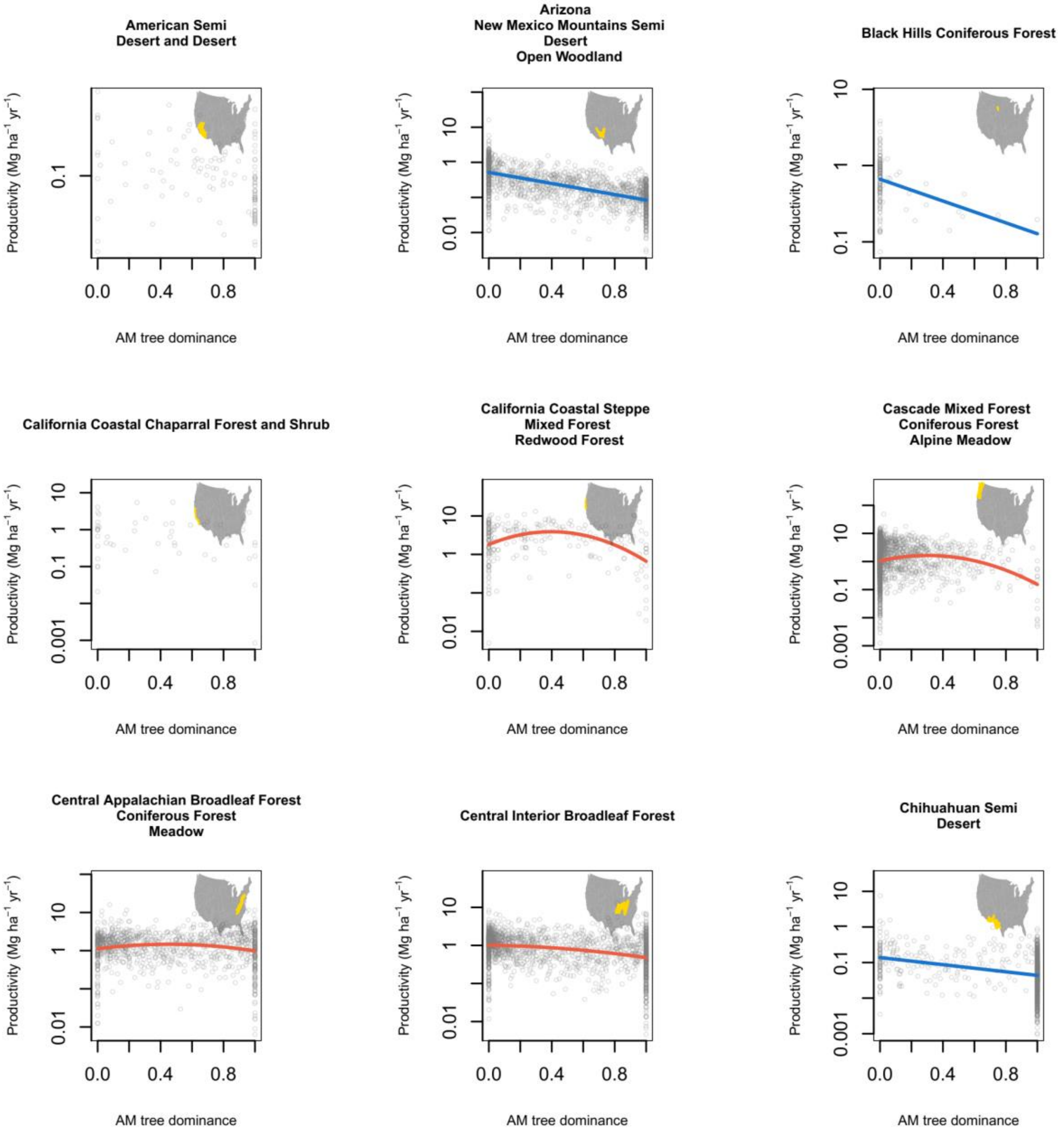
American Semi Desert and Desert
Arizona New Mexico Mountains Semi Desert Open Woodland
Black Hills Coniferous Forest
California Coastal Chaparral Forest and Shrub
California Coastal Steppe Mixed Forest Redwood Forest
Cascade Mixed Forest Coniferous Forest Alpine Meadow
Central Appalachian Broadleaf Forest Coniferous Forest Meadow
Central Interior Broadleaf Forest
Chihuahuan Semi Desert
Productivity (Mg ha⁻¹ yr⁻¹)
AM tree dominance
0.0
0.4
0.8
0.001
0.01
0.1
1
10

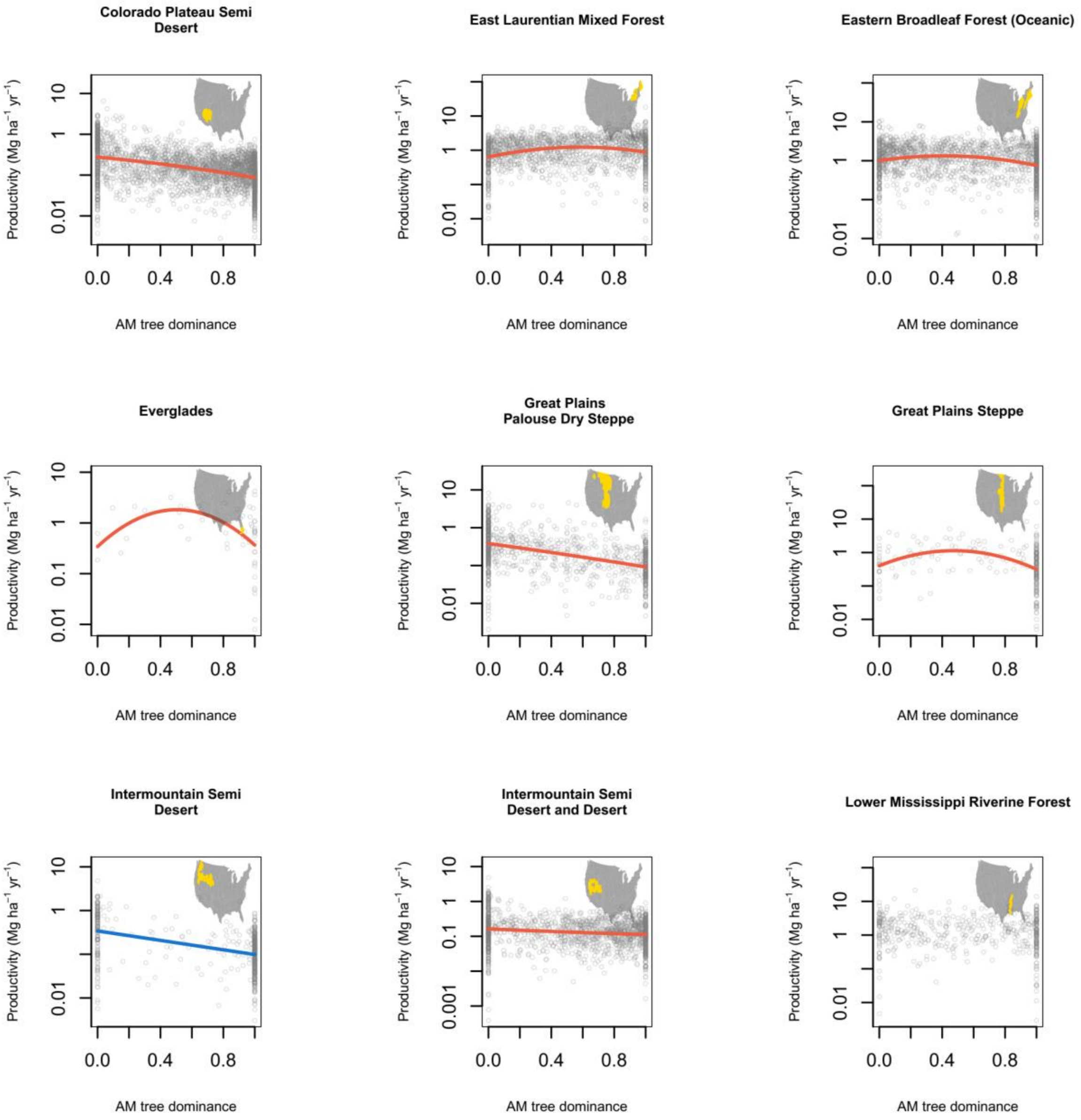
Colorado Plateau Semi Desert
East Laurentian Mixed Forest
Eastern Broadleaf Forest (Oceanic)
Everglades
Great Plains Palouse Dry Steppe
Great Plains Steppe
Intermountain Semi Desert
Intermountain Semi Desert and Desert
Lower Mississippi Riverine Forest
Productivity (Mg ha⁻¹ yr⁻¹)
AM tree dominance

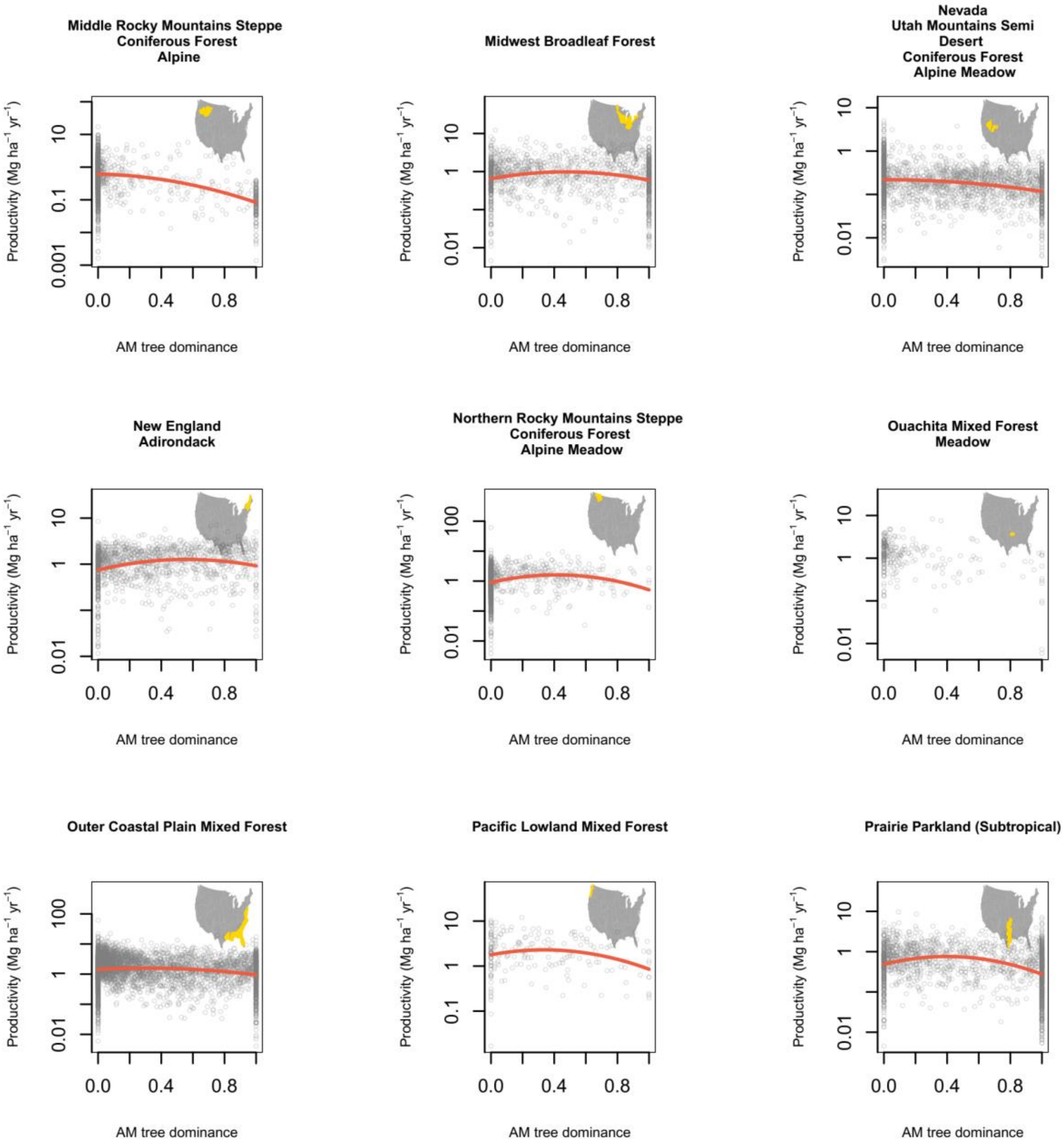
Middle Rocky Mountains Steppe
Coniferous Forest
Alpine
Midwest Broadleaf Forest
Nevada
Utah Mountains Semi
Desert
Coniferous Forest
Alpine Meadow
New England
Adirondack
Northern Rocky Mountains Steppe
Coniferous Forest
Alpine Meadow
Ouachita Mixed Forest
Meadow
Outer Coastal Plain Mixed Forest
Pacific Lowland Mixed Forest
Prairie Parkland (Subtropical)
Productivity (Mg ha^{-1} yr^{-1})
AM tree dominance
0.0
0.4
0.8

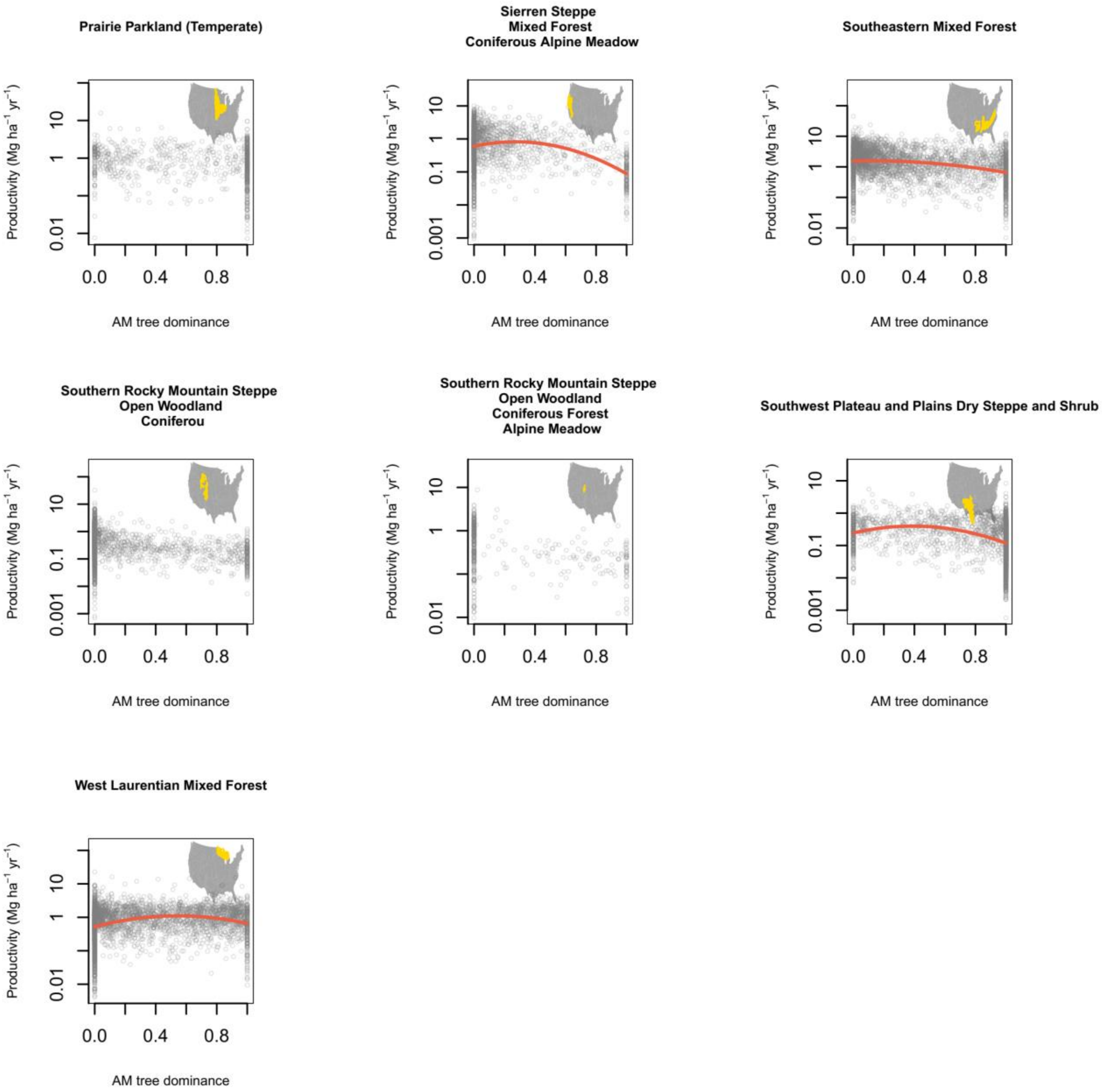

Supplementary Fig. S15 | Ecoregion-level relationships between AM tree dominance and productivity for plots with five or fewer species. The colored area in the inset map indicates the distribution of the corresponding ecoregion. See Supplementary Data 3 for statistical results.

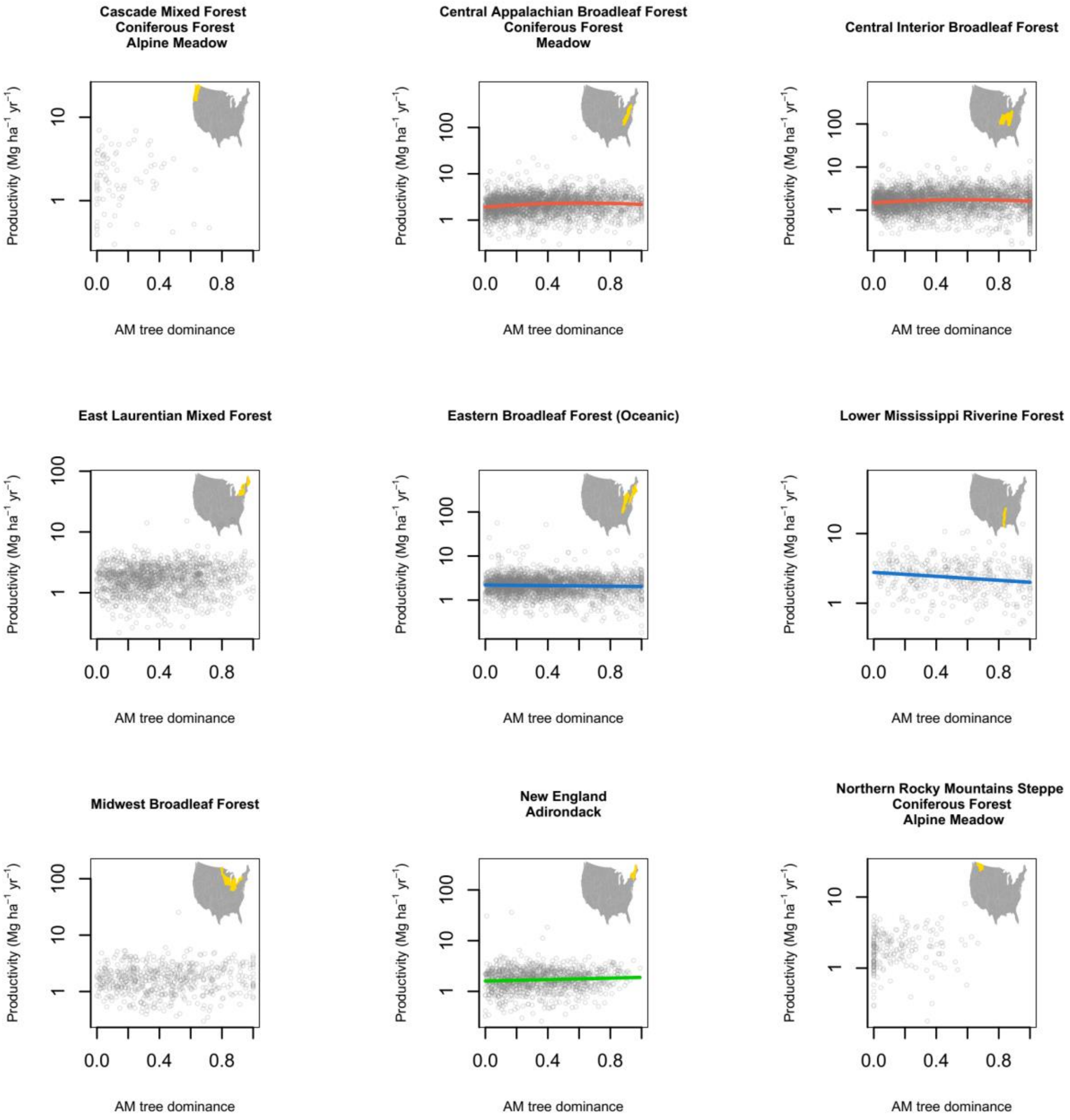
Cascade Mixed Forest
Coniferous Forest
Alpine Meadow
Central Appalachian Broadleaf Forest
Coniferous Forest
Meadow
Central Interior Broadleaf Forest
East Laurentian Mixed Forest
Eastern Broadleaf Forest (Oceanic)
Lower Mississippi Riverine Forest
Midwest Broadleaf Forest
New England
Adirondack
Northern Rocky Mountains Steppe
Coniferous Forest
Alpine Meadow
Productivity (Mg ha^{-1} yr^{-1})
AM tree dominance
0.0
0.4
0.8
1
10
100

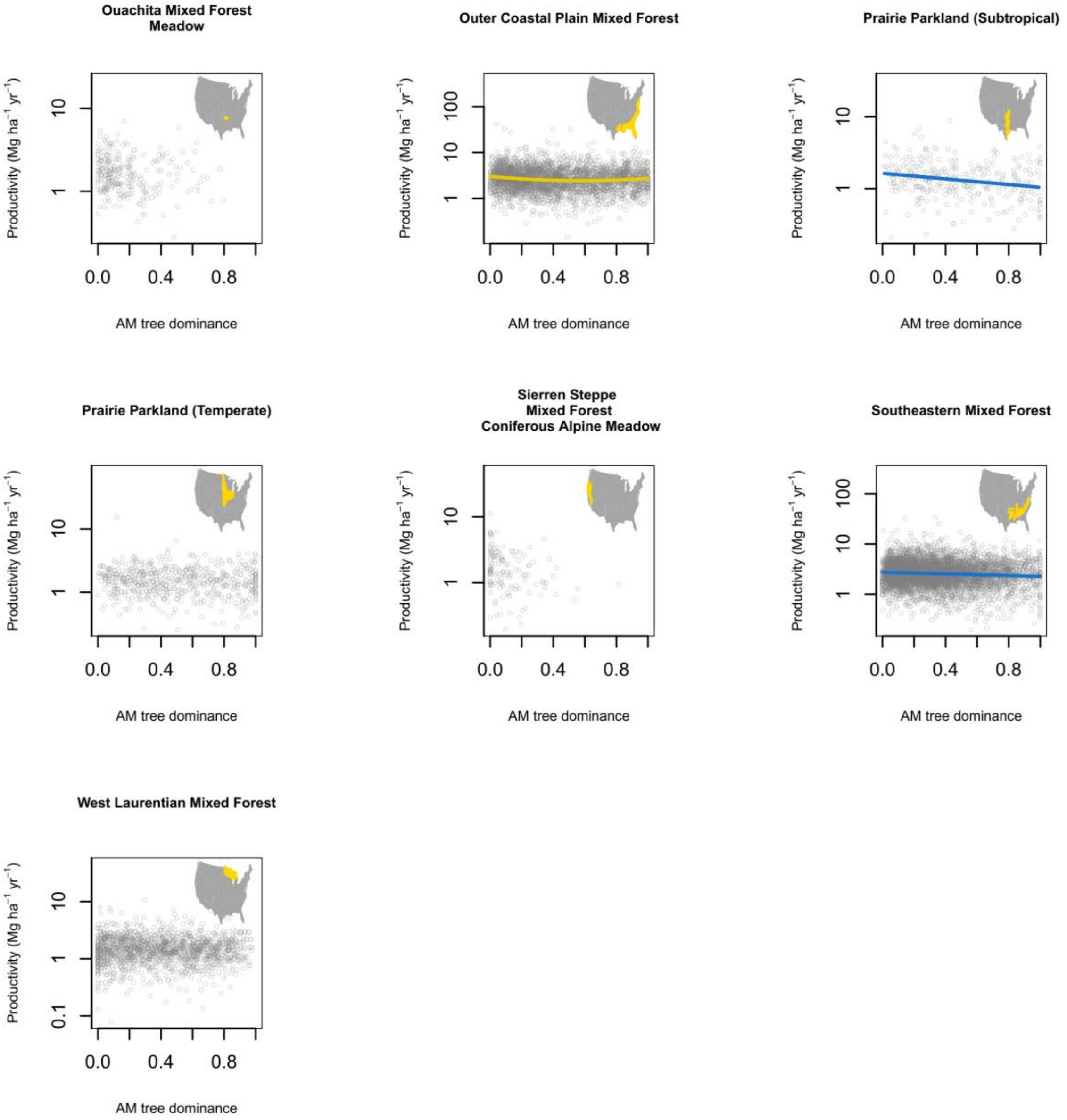

Supplementary Fig. S16 | Ecoregion-level relationships between AM tree dominance and productivity for plots with more than five species. The colored area in the inset map indicates the distribution of the corresponding ecoregion. See Supplementary Data 4 for statistical results.
