# Supplementary material for: Higher productivity in forests with mixed mycorrhizal strategies
Source: Nat Commun. 2023 Mar 13;14:1377. doi: 10.1038/s41467-023-36888-0 (PMC10011551; doi:10.1038/s41467-023-36888-0)
Supplement: Supplementary file 6 — Reporting Summary [file 41467_2023_36888_MOESM6_ESM.pdf]

nature portfolio

Corresponding author(s): Shan Luo

Last updated by author(s): Feb 10, 2023

Reporting Summary

Nature Portfolio wishes to improve the reproducibility of the work that we publish. This form provides structure for consistency and transparency in reporting. For further information on Nature Portfolio policies, see our Editorial Policies and the Editorial Policy Checklist.

Statistics

For all statistical analyses, confirm that the following items are present in the figure legend, table legend, main text, or Methods section.

n/a	Confirmed	
☐	☒	The exact sample size (*n*) for each experimental group/condition, given as a discrete number and unit of measurement
☐	☒	A statement on whether measurements were taken from distinct samples or whether the same sample was measured repeatedly
☐	☒	The statistical test(s) used AND whether they are one- or two-sided<br>*Only common tests should be described solely by name; describe more complex techniques in the Methods section.*
☐	☒	A description of all covariates tested
☐	☒	A description of any assumptions or corrections, such as tests of normality and adjustment for multiple comparisons
☐	☒	A full description of the statistical parameters including central tendency (e.g. means) or other basic estimates (e.g. regression coefficient) AND variation (e.g. standard deviation) or associated estimates of uncertainty (e.g. confidence intervals)
☐	☒	For null hypothesis testing, the test statistic (e.g. *F*, *t*, *r*) with confidence intervals, effect sizes, degrees of freedom and *P* value noted<br>*Give P values as exact values whenever suitable.*
☒	☐	For Bayesian analysis, information on the choice of priors and Markov chain Monte Carlo settings
☒	☐	For hierarchical and complex designs, identification of the appropriate level for tests and full reporting of outcomes
☐	☒	Estimates of effect sizes (e.g. Cohen's *d*, Pearson's *r*), indicating how they were calculated

Our web collection on statistics for biologists contains articles on many of the points above.

Software and code

Policy information about availability of computer code

Data collection	No software was used for collecting forest inventory data. The software R (v.4.0.3) was used for collecting climatic and soil data.
Data analysis	All statistical analyses were conducted using the software R (v.4.0.3) and the following packages: data.table, ggplot2, ggpubr, ggspatial, raster, dplyr, lme4, piecewiseSEM. R codes that support the findings are available at Figshare with the identifier https://doi.org/10.6084/m9.figshare.22060238

For manuscripts utilizing custom algorithms or software that are central to the research but not yet described in published literature, software must be made available to editors and reviewers. We strongly encourage code deposition in a community repository (e.g. GitHub). See the Nature Portfolio guidelines for submitting code & software for further information.

Data

Policy information about availability of data

All manuscripts must include a data availability statement. This statement should provide the following information, where applicable:

- Accession codes, unique identifiers, or web links for publicly available datasets
- A description of any restrictions on data availability
- For clinical datasets or third party data, please ensure that the statement adheres to our policy

The data that support the findings of this study are available at https://doi.org/10.6084/m9.figshare.22060238. Original FIA data are available at https://apps.fs.usda.gov/fia/datamart/datamart.html. Climate data are available at the Global Climate Data-WorldClim (www.worldclim.org/). Soil pH data are available at GSDE (http://globalchange.bnu.edu.cn/research/soilw). The raw CFI dataset used for the Supplementary Fig. S12 are unpublished, because another manuscript is in

preparation. But we have provided source data for Supplementary Fig. S12.

Human research participants

Policy information about studies involving human research participants and Sex and Gender in Research.

Reporting on sex and gender	N/A
Population characteristics	N/A
Recruitment	N/A
Ethics oversight	N/A

Note that full information on the approval of the study protocol must also be provided in the manuscript.

Field-specific reporting

Please select the one below that is the best fit for your research. If you are not sure, read the appropriate sections before making your selection.

☐ Life sciences ☐ Behavioural & social sciences ☒ Ecological, evolutionary & environmental sciences

For a reference copy of the document with all sections, see nature.com/documents/nr-reporting-summary-flat.pdf

Ecological, evolutionary & environmental sciences study design

All studies must disclose on these points even when the disclosure is negative.

Study description	Our study examines how forest mycorrhizal composition (dominance of one mycorrhizal strategy versus co-dominance, i.e. mixture of two mycorrhizal strategies) influences forest productivity and whether these processes are related to tree taxonomic diversity, across the contiguous USA. No manipulative field/lab experiment was performed.
Research sample	Our research sample is a database of 74,563 forest inventory plots that span 35 eco-regions across the contiguous USA, including information about tree species richness, forest aboveground biomass productivity, and tree mycorrhizal composition. All plots have accompanying climatic and soil variables as covariates. The sample is believed to cover broad environmental gradients.
Sampling strategy	We did not predetermine a sample size. We included all available plots except that with missing values. Our final dataset includes 74,563 plots.
Data collection	We (S. Luo & I. Jo) downloaded forest inventory data from the FIA program (https://apps.fs.usda.gov/fia/datamart/datamart.html); S. Luo downloaded the climate data from WorldClim (www.worldclim.org/) and soil pH data from GSDE (http://globalchange.bnu.edu.cn/research/soilw). S. Luo and I. Jo complied the data using basic functions in software R .
Timing and spatial scale	The spatial scale of our study is regional. Timing scale is not applicable.
Data exclusions	We excluded plots where productivity equals zero and that with missing environmental variables and mycorrhizal assignment.
Reproducibility	All analyses are reproducible with provided dataset and R code.
Randomization	No randomization was used when collecting the data. We included all observations in our dataset, except plots with missing values.
Blinding	Not applicable.

Did the study involve field work? ☐ Yes ☒ No

Reporting for specific materials, systems and methods

We require information from authors about some types of materials, experimental systems and methods used in many studies. Here, indicate whether each material, system or method listed is relevant to your study. If you are not sure if a list item applies to your research, read the appropriate section before selecting a response.

Materials & experimental systems

n/a	Involved in the study
☒	☐ Antibodies
☒	☐ Eukaryotic cell lines
☒	☐ Palaeontology and archaeology
☒	☐ Animals and other organisms
☒	☐ Clinical data
☒	☐ Dual use research of concern

Methods

n/a	Involved in the study
☒	☐ ChIP-seq
☒	☐ Flow cytometry
☒	☐ MRI-based neuroimaging
