# Supplementary material for: Higher productivity in forests with mixed mycorrhizal strategies
Source: Nat Commun. 2023 Mar 13;14:1377. doi: 10.1038/s41467-023-36888-0 (PMC10011551; doi:10.1038/s41467-023-36888-0)
Supplement: Supplementary file 8 — Description of Additional Supplementary Files [file 41467_2023_36888_MOESM8_ESM.pdf]

Description of Additional Supplementary Information

File name: Supplementary Data 1

Description: Statistical results for the effects of AM proportion on productivity across all plots in linear and quadratic models.

File name: Supplementary Data 2

Description: Statistical results for the effects of ECM proportion on productivity across all plots in linear and quadratic models.

File name: Supplementary Data 3

Description: Linear and quadratic model results for the effects of AM proportion on productivity in plots with five or less species.

File name: Supplementary Data 4

Description: Linear and quadratic model results for the effects of AM proportion on productivity in plots with more than five species.
